# Phytochemical characterization and effects of *Dolomiaea costus* root extract on ovariectomy-induced metabolic, skeletal, and uterine alterations in rats

**DOI:** 10.3389/fphar.2026.1841164

**Published:** 2026-10-09

**Authors:** Tahany Saleh Aldayel, Hany Salah Mahmoud, Heba M. A. Abdelrazek, Doaa H. Elsayed, Mona A. Higo, Enas E. Eltamany, Heba Nageh Gad EL-Hak

**Affiliations:** 1 Department of Health Sciences, Clinical Nutrition, College of Health and Rehabilitation Sciences, Princess Nourah bint Abdulrahman University, Riyadh, Saudi Arabia; 2 Center of Scientific Foundation for Experimental Studies and Research, Ismailia, Egypt; 3 Department of Physiology, Faculty of Veterinary Medicine, Suez Canal University, Ismailia, Egypt; 4 Department of Theriogenology, Faculty of Veterinary Medicine, Suez Canal University, Ismailia, Egypt; 5 Department of Zoology, Faculty of Science, Al-Arish University, North Sinai, Egypt; 6 Department of Pharmacognosy, Faculty of Pharmacy, Suez Canal University, Ismailia, Egypt; 7 Department of Zoology, Faculty of Sciences, Suez Canal University, Ismailia, Egypt

**Keywords:** Asteraceae, bone alterations, *Dolomiaea costus*, estrogen receptor α, metabolic dysfunction, ovariectomy

## Abstract

**Background:**

Estrogen deficiency following ovariectomy produces metabolic, skeletal, and uterine alterations.

**Methods:**

In this study, we evaluated an authenticated and chemically characterized dichloromethane–methanol (1:1, v/v) root extract of *Dolomiaea costus* (Syn. *Saussurea costus*) in ovariectomized female Wistar rats. Thirty rats were assigned to sham-operated, ovariectomized untreated, and ovariectomized extract-treated groups. Beginning 3 weeks after surgery, the treated group received the extract orally at 300 mg/kg body weight/day for 12 weeks. Metabolic and lipid variables, adipokines, antioxidant status, and bone-related biomarkers were determined. Uterine, femoral, hepatic, and adipose tissues were examined histologically, while estrogen receptor-α (ER-α) and tumor necrosis factor-α (TNF- α) immunoreactivity were evaluated. The extract was characterized using spectrophotometric assays, GC–MS, and HPLC-DAD.

**Results:**

Ovariectomy was associated with increased body and adipose-tissue mass, dyslipidemia, impaired glucose homeostasis, altered adipokine concentrations, decreased antioxidant capacity, and structural alterations in the uterus, bone, liver, and adipose tissue. Compared with untreated ovariectomized rats, extract administration was associated with improvements in several biochemical and histological outcomes and with increased uterine ER-α immunoreactivity. Chemical profiling indicated the presence of sesquiterpene lactones, fatty-acid derivatives, and phenolic metabolites.

**Conclusion:**

Increased ER-α immunoreactivity is consistent with an estrogen-responsive effect but does not establish direct estrogen-receptor agonism. These preclinical findings support further standardized pharmacological and toxicological evaluation of the extract; they do not establish efficacy or safety in menopausal women.

## 1 Introduction

Menopause is defined as the permanent cessation of menstruation following the loss of ovarian follicles (Sherman, 2005). This transition can be prematurely induced by follicle-depleting factors such as tobacco use or surgical intervention. Rather than originating centrally, menopause is fundamentally a state of ovarian failure, which subsequently disrupts the hypothalamic–pituitary–gonadal feedback loop and alters the secretion of regulatory hormones (Naftolin et al., 2019). As follicular reserves decline, the ovaries become unresponsive to gonadotropins such as follicle-stimulating hormone (FSH) and LH, leading to a marked reduction in estrogen and progesterone synthesis (Zhu et al., 2023). Despite this decline, low levels of circulating estrogens persist in postmenopausal women, largely due to extragonadal aromatization of androgens primarily in adipose tissue, where ovarian and adrenal precursors are converted into estrogen (Kuryłowicz, 2023).

Due to ovarian failure and the loss of negative feedback, FSH levels rise during menopause, as the ovarian–hypothalamic–pituitary axis remains functional (Barnhart and Osheroff, 1998). The decline in estrogen and inhibin production, particularly from granulosa cells, leads to reduced inhibin levels and elevated FSH, a key marker of menopause (Dumesic et al., 2015). Since women rely solely on their ovaries for oocytes, which are also the primary source of estrogen, progesterone, and a major contributor to androgens, the depletion of oocytes leads to infertility (Walters, 2015).

The main clinical consequence of halted progesterone production is an elevated risk for endometrial pathologies, including proliferation, hyperplasia, and cancer. This risk arises from exposure to unopposed estrogen, whether from endogenous production or exogenous hormone therapy in menopausal women (Genazzani et al., 2021).

Most menopausal symptoms stem from estrogen deficiency, including vasomotor symptoms, urogenital atrophy, osteoporosis, cardiovascular disease, cancer, psychiatric disturbances, and cognitive decline (Crandall et al., 2023). Osteoporosis often leads to musculoskeletal issues such as back pain, fractures from minor trauma, height loss, and reduced mobility (Sinaki, 1998). In recent years, postmenopausal obesity, genital atrophy, and osteoporosis have emerged as major global health concerns due to their increasing prevalence (van Dijk et al., 2015).

*Dolomiaea costus (*Syn*. Saussurea costus)* is a traditional treatment that has been successfully used for reducing menopausal symptoms and conditions for thousands of years (Ali et al., 2021). Moreover, *D. costus* is a medicinal species whose dried root is used as a botanical drug in several traditional medical systems. The root has principally been reported for gastrointestinal complaints, including abdominal discomfort, distension, impaired digestion, and diarrhea, and it has also been used in preparations intended for respiratory and inflammatory conditions (Mishra et al., 2022). Direct traditional use specifically for menopausal disorders has not been clearly established. Therefore, the rationale for the present investigation is pharmacological rather than the direct validation of a traditional menopausal indication. Chemically, *D. costus* root is characterized predominantly by sesquiterpene lactones and related terpenoid metabolites, including costunolide and dehydrocostus lactone (Zhang et al., 2025). Phenolic acids and flavonoid metabolites have also been reported. These metabolite classes have been associated in experimental studies with antioxidant, inflammatory, and metabolic activities (Gasmi et al., 2022). Nevertheless, the composition and biological activity of botanical extracts depend strongly on plant origin, processing, and extraction conditions. Consequently, characterization of the specific extract batch used in an experimental study is required.

Although *D. costus* root extracts have previously been investigated for antioxidant, anti-inflammatory, and protective effects in several experimental settings (AL-Alh et al., 2024; Lodh and Chakraborty, 2024), evidence concerning their effects in estrogen-deficient models remains limited. Moreover, to date, no study has provided an integrated evaluation of *D. costus* in an ovariectomized model combining metabolic profiling, lipid regulation, adipokine status, skeletal morphology, hepatic histology, uterine estrogen receptor-α (ER- α) immunoreactivity, adipose inflammatory activity, and systemic antioxidant capacity alongside detailed phytochemical characterization. This multidimensional approach may provide a broader assessment of the physiological alterations associated with ovarian-hormone deficiency and their possible modification by the extract. Leptin, adiponectin, and resistin were selected because ovarian-hormone deficiency is accompanied by changes in adipose-tissue mass and endocrine function. TNF-α was assessed as an indicator of adipose inflammatory activity, whereas total antioxidant capacity was used as a systemic measure related to redox balance. Uterine ER-α immunoreactivity was evaluated because uterine tissue is strongly estrogen-responsive. Calcium-related variables and femoral morphology were included to assess selected skeletal consequences of estrogen deficiency. These endpoints were interpreted as complementary indicators and not as direct measurements of untested intracellular signaling pathways. Therefore, in the present study, we aimed to evaluate the effects of a chemically characterized *D. costus* root extract on selected metabolic, adipokine, antioxidant, skeletal, hepatic, adipose, and uterine alterations in ovariectomized rats. The present study was, therefore, designed as an integrated preclinical assessment of these outcomes in ovariectomized rats. The study does not establish clinical efficacy or identify a definitive molecular mechanism. In the present study, we aimed to evaluate the effects of a chemically characterized *D. costus* root extract on selected metabolic, skeletal, hepatic, adipose, and uterine alterations in ovariectomized rats and examine possible associations with their measured chemical profile.

## 2 Materials and methods

### 2.1 Animals

Thirty healthy female Wistar rats (100 and 110 g) were acquired from the Suez Canal University, Faculty of Veterinary Medicine’s laboratory animal house on 2 July 2024. Rats were allowed free access to a basal diet for 2 weeks prior to the experiment as part of their acclimatization. Water was always available. This study was conducted in compliance with international guidelines for the ethical use of animals in research. All experimental protocols were reviewed and approved by the Ethical Research Committee at the Faculty of Science, Suez Canal University (Approval No. REC332/2024).

### 2.2 Experimental procedure

Ten female rats underwent a sham procedure under general anesthesia induced by intravenous injection of thiopental sodium at a dose of 30 mg/kg body weight, in accordance with the guidelines of the American Veterinary Medical Association (AVMA). The onset of anesthesia was typically observed within 30 s–60 s post-injection. Adequate depth of anesthesia was confirmed by loss of pedal withdrawal and corneal reflexes. Twenty additional female rats underwent bilateral ovariectomy (OVX) under the same anesthetic protocol. The procedure was performed through a midline abdominal incision as described by Lasota and Danowska-Klonowska (2004). During surgery, anesthesia was maintained with supplemental doses of thiopental sodium (5–10 mg/kg, IV) as needed. Following surgery, all animals received an oral suspension of amoxicillin at a dose of 10 mg/kg body weight once daily for three consecutive days, as described by Helmy et al. (2014). Three weeks after ovariectomy, eligible ovariectomized rats were allocated to two equal groups using a computer-generated random sequence. The allocation sequence was generated by an investigator who was not involved in outcome assessment. Group codes were assigned to the animals, biological samples, and tissue slides using coded identifiers. The investigators conducting biochemical assays, histological measurements, immunohistochemical assessment, and image analysis were blinded to group identity until data collection and statistical analysis were completed. The investigator administering the treatments was aware of group allocation because the treatment formulations differed. The OVX group received the dosing vehicle, whereas the OVX/*D* group received the root extract orally at 300 mg/kg body weight/day for 12 weeks (Elshaer et al., 2022). The daily dose of 300 mg/kg body weight was selected based on previous rat studies using orally administered *D. costus* root extracts (Elshaer et al., 2022). Using body-surface-area scaling, a rat dose of 300 mg/kg corresponds approximately to a human-equivalent dose of 48.6 mg/kg, or approximately 2.9 g for a 60-kg adult. This calculation is only a preliminary scaling estimate and is not a recommended clinical dose. Body-surface-area conversion does not account for extract standardization, absorption, metabolism, pharmacokinetics, toxicity, or species differences. The differences in the experimental models and extract preparations mean that this dose should be regarded as an exploratory pharmacological dose rather than an established optimal dose for estrogen-deficiency-related outcomes. The sham-operated group received an equivalent volume of the dosing vehicle. No estradiol-, raloxifene-, or hormone-therapy-treated positive-sham group was included because the original experiment was designed to determine whether the extract modified selected ovariectomy-associated outcomes relative to untreated ovariectomized animals. Consequently, the study cannot establish comparative efficacy against standard estrogenic or anti-osteoporotic treatments. Thirty rats entered and completed the experiment, with 10 animals in each group. Biochemical outcomes were measured in all 10 animals per group. Six animals per group were randomly selected for histomorphometric and immunohistochemical analyses, with three sections examined per animal. Section-level measurements were averaged to produce a single value for each animal. At the end of the experimental period, animals were euthanized by intravenous administration of an overdose of thiopental sodium (>100 mg/kg body weight), in accordance with AVMA guidelines. Death was confirmed by cessation of respiration and heartbeat.

### 2.3 Botanical material: authentication and extraction

Commercial dried roots of *D. costus* (Falc.) Kasana and A.K. Pandey (Asteraceae; synonym: *Saussurea costus* (Falc.) Lipsch.) were purchased from the Prophetic Medicine Foundation, Ismailia, Egypt, Commercial Registration No. 6332001/11133316, in July 2022.

The botanical material was authenticated by Dr/Yassmine Ahmed Hassan, Associate Professor of Plant Taxonomy, Department of Botany and Microbiology, Faculty of Science, Suez Canal University, Ismailia, Egypt. Authentication was based on macroscopic and organoleptic examination of the dried roots, including their shape, dimensions, external color, surface characteristics, fracture, odor, and taste, along with comparison with an authenticated reference specimen and the description of Aucklandiae Radix in the Chinese Pharmacopoeia, 2020 edition, volume I.

The accepted scientific name and synonym were verified using Plants of the World Online, Royal Botanic Gardens, Kew. A voucher specimen has been deposited in the Pharmacognosy Department Herbarium, Faculty of Pharmacy, Suez Canal University, Ismailia, Egypt, under registration number of S.c-2022-1.

A single authenticated batch of dried *D. costus* roots was ground using a laboratory mill and passed through a 40-mesh (particle size ≤425 µm) sieve. Five hundred grams of the powdered material were macerated three times with dichloromethane–methanol (1:1, *v/v*; 750 mL per extraction cycle) for 48 h per cycle at 25 °C, with intermittent agitation (manual shaking every 8 h–12 h). The cumulative solvent volume was 2,250 mL. After each cycle, the extract was filtered through Whatman No. 1 filter paper, and the three filtrates (after checking their consistency by TLC analysis) were pooled together. The combined filtrates were dried under reduced pressure of approximately 150 mbar–175 mbar at 40 °C. A total of 27 g of dry extract was obtained, corresponding to an extraction yield of 5.4% *w/w* and a native drug-to-extract ratio of approximately 18.5:1. The extract was assigned the internal laboratory batch code DCR-2022-01, transferred to airtight amber-glass containers, and stored at 4 °C, and they were protected from light and moisture until chemical analysis and animal administration. The stability of the extract was assured by TLC analysis for monitoring any changes in chromatographic fingerprints, compound degradation, and spot separation. For oral dosing, the dry extract was suspended in normal saline (0.9% NaCl) at a concentration of 30 mg/mL and administered at a volume of 10 mL/kg body weight once daily for 12 consecutive weeks, delivering a dose of 300 mg/kg body weight. The formulation was prepared freshly each day immediately before dosing and maintained at room temperature, protected from light, for the brief interval between preparation and gavage. The OVX vehicle-control group received an equivalent volume (10 mL/kg) of saline alone.

### 2.4 Total phenolic and total flavonoids contents of *Dolomiaea costus* root extract

The extract’s total phenolic content (TPC) was determined via the Folin–Ciocalteu method (Lamuela-Raventós, 2018). This assay quantifies phenolics by measuring the blue complex formed from their redox reaction with the reagent in a sodium carbonate solution. The resulting chromophore’s absorbance intensity, measured spectrophotometrically, exhibits a linear relationship with the phenolic concentration, enabling quantitative determination of TPC expressed as gallic acid equivalents. The quantification of TPC was conducted spectrophotometrically. A Milton Roy Spectronic 1201 spectrophotometer was used to measure absorbance at 630 nm, and a gallic acid calibration standard allowed for the expression of results in mg of gallic acid equivalents per gram of dry extract (mg GAE/g). Meanwhile, the total flavonoid content (TFC) was assessed spectrophotometrically using the aluminum chloride (AlCl_3_) method, following the procedure outlined by Shraim et al. (2021). This method depends on the interaction of AlCl_3_ with the carbonyl (C-4) and the hydroxyl (either in C-3 or C-5) groups in the flavonoid nucleus forming a stable complex. In addition, the ortho-dihydroxyl groups in the A- or B-ring of flavonoids produces acid-unstable adducts with AlCl_3_, leading to UV bathochromic shifts. Quercetin is usually used as a standard in this method. Using a Milton Roy Spectronic 1201 spectrophotometer, the TFC was determined at 510 nm and quantified as mg quercetin equivalents per gram of dry extract (mg QE/g).

### 2.5 Estimation of total terpenoids content in crude extract of *Dolomiaea costus* root

The total terpenoids content (TTC) in *D. costus* crude extract was quantified using the Salkowski colorimetric assay according to Mammate et al. (2023). The method involves mixing the extract with chloroform, followed by addition of concentrated H_2_SO_4_ to form a reddish-brown precipitate with a chromogenic supernatant, the intensity of which correlates with the terpenoid concentration. For terpenoid quantification, the supernatant was collected and analyzed spectrophotometrically at 538 nm using a Milton Roy Spectronic 1201 instrument (Houston, TX, USA). Linalool served as the reference standard, with the total terpenoid content expressed as linalool equivalents (mg/g dry weight). All measurements were conducted in triplicate to ensure analytical precision.

### 2.6 Chemical antioxidant-capacity assays

The Milton Roy Spectronic 1201 UV/Vis spectrophotometer (USA) served as the central analytical instrument for all antioxidant assays. Because antioxidant activity is mechanism- and assay-dependent, and because natural products are complex and have reaction modes as antioxidants whether by SET, HAT, or transition-metal chelation (Eltamany et al., 2022), and several complementary chemical assays were used to characterize the chemical antioxidant properties of the extract. The DPPH and ABTS assays evaluate free-radical-scavenging activity under different reaction conditions, whereas the FRAP assay assesses the electron-donating and ferric-reducing capacity. The phosphomolybdenum assay provides an additional estimate of the overall reducing capacity of the extract. No single assay comprehensively represents all antioxidant mechanisms; therefore, the combined assays were used to provide a broader chemical characterization of the extract’s chemical antioxidant activity.

#### 2.6.1 DPPH free radical scavenging activity

The DPPH activity of *D*. *costus* crude extract was evaluated according to the method described by Binobead et al. (2024). Triplicate absorbance readings at 515 nm were recorded against a blank reference. Ascorbic acid served as the reference standard. The percentage of DPPH radical inhibition (PI) was calculated using the following equation:

$$\text{PI}=\left[\;\left(\text{AC}-\text{AT}\right)/\text{AC}\times 100\right],$$

where AC represents the absorbance of the control and AT represents the absorbance of the test sample; the chemical half-maximal inhibitory concentration (IC_50_) was determined from the dose–response curve generated using GraphPad Prism software (San Diego, CA, USA).

#### 2.6.2 ABTS radical-cation scavenging assay

The ABTS capacity of *D*. *costus* crude extract against ABTS•+ radicals were assessed according to the protocol established by Hussien et al. (2024). The assay measured the extract’s capacity to neutralize 2,2-azino-bis (3-ethylbenzothiazoline-6-sulfonic acid) radical cations. The ABTS•+ radical scavenging assay was performed with butylhydroxytoluene (BHT) as the positive control. Triplicate absorbance readings (734 nm) were recorded against blank references. Radical scavenging activity was calculated as the percentage of DPPH radical inhibition using the formula:

$$\text{Inhibition }\left(\%\right)=100\times \left[\left(\text{Acontrol}-\text{Asample}\right)\;/\text{ Acontrol}\right],$$

where Acontrol is the absorbance of the control reaction and Asample is the absorbance of the test sample. The chemical IC_50_ was then determined by analyzing the dose–response curves with GraphPad Prism software.

#### 2.6.3 Ferric-reducing antioxidant power (FRAP) assay

The FRAP of *D*. *costus* crude extract was determined as described in Eltamany et al. (2020), which measures the reduction of ferricyanide ions in a concentration-dependent manner. Absorbance readings at 700 nm were taken in triplicate versus a blank. With butylated hydroxytoluene (BHT) as the standard, the results were quantified as mM Fe^2+^ equivalents per gram of dry sample (mM Fe^2+^/g).

#### 2.6.4 Chemical phosphomolybdenum reducing-capacity assay

The chemical reducing capacity of the extract was evaluated using the phosphomolybdenum method. In this assay, antioxidant constituents reduce Mo (VI) to Mo (V) under acidic conditions, leading to the formation of a green phosphomolybdate complex. Absorbance was measured at 695 nm. Triplicate measurements were evaluated using a gallic acid calibration curve, and the results were expressed as milligrams of gallic acid equivalents per gram of dry extract. This cell-free assay evaluates the reducing capacity of the extract and is distinct from the serum total antioxidant capacity measured in the experimental animals (Genwali et al., 2013).

### 2.7 Chemical profiling of *Dolomiaea costus* extract

#### 2.7.1 Gas chromatography mass spectrometry (GC–MS) analysis of the lipoidal matter of *Dolomiaea costus* extract

##### 2.7.1.1 Preparation of *Dolomiaea costus* hexane fraction (lipoidal matter)

The defatting process of *D. costus* crude extract (10 g) was performed by suspension in 70% methanol (100 mL) followed by liquid–liquid partitioning with hexane (3 × 50 mL). The pooled hexane fractions were evaporated under reduced pressure to yield the hexane-soluble fraction. The remaining defatted methanol extract was then concentrated *in vacuo* to obtain the defatted *D. costus* extract (Oyetayo et al., 2025).

##### 2.7.1.2 Preparation of the saponified and unsaponified parts of *Dolomiaea costus* hexane fraction

Preparation of the saponified and unsaponified parts of *D. costus* hexane fraction was achieved following the protocol of Prieto Vidal et al. (2018). The hexane-extracted material (2 g) underwent saponification via refluxing with 15 mL of 10% alcoholic KOH (5 h, 100 °C). After dilution of the cooled reaction mixture with 20 mL distilled H_2_O, unsaponifiable constituents underwent triple liquid–liquid extraction using diethyl ether (20 mL × 3). After washing the pooled ether fractions to a neutral pH, they were dried over anhydrous Na_2_SO_4_, filtered, and concentrated under reduced pressure at 40 °C for later GC–MS analysis. The aqueous phase was acidified (10% HCl) to pH ≤ 2, liberating fatty acids, which were similarly extracted with diethyl ether (3 × 20 mL). After dehydration (Na_2_SO_4_) and solvent evaporation under vacuum, the saponifiable fraction was esterified via methanolysis: refluxing the residue with 50 mL anhydrous methanol containing 3 mL conc. H_2_SO_4_ (2 h, 100 °C). Fatty acid methyl esters were extracted with diethyl ether (3 × 20 mL), dried over anhydrous Na_2_SO_4_, and solvent-evaporated to constant mass.

##### 2.7.1.3 Conditions for GC–MS analysis of lipoidal constituents

GC–MS analysis was conducted at the Drug Discovery Research and Development Center, Faculty of Pharmacy, Ain Shams University, Cairo, using the HS/GC–MS (Shimadzu QP2020, Japan) system with (split/split-less injector) with a fused-silica RTX-1MS capillary column (30 m × 0.25 mm i.d. × 0.25 µm thickness). The operating conditions for analyzing the saponifiable and unsaponifiable fractions were adjusted according to Hasan et al. (2026). Putative compounds were assigned by matching their spectral data with NIST17 s.lib mass spectral library (match threshold >80%) using the retention index (data where applicable) to support the identification. The results indicate only volatile and semi-volatile compounds in *D*. *costus* since the extract was analyzed without derivatization (Ullah et al., 2026). Although comparison with authentic standards would confirm the compound identification, matching with available reference databases, together with mass spectral data, is an acceptable approach for compound identification in the phytochemical analysis (Hasan et al., 2026) offering a second level of compound identification according to Schymanski et al. (2015). The percentage of each compound was determined by GC–MS solution software® using the percentage peak area method by determining the area of the target compound peak as a proportion of the total area of all the peaks detected (Saeed Kotb et al., 2020). Peak-area normalization was used to estimate the relative abundance and was not considered an absolute quantitative analysis.

#### 2.7.2 HPLC-DAD analysis of *Dolomiaea costus* extract

##### 2.7.2.1 Reference standards

The qualitative analysis of *D. costus* extract was performed using 10 common phenolic reference standards, including four phenolic acids (caffeic, chlorogenic, gallic, and ellagic acids) and six flavonoids (catechin, hesperidin, rutin, quercetin, kaempferol, and apigenin), all of which were obtained as authentic standards (≥98% purity) from Nawah Scientific Inc. (Cairo, Egypt).

##### 2.7.2.2 Apparatus

For the phytochemical analysis, separation and identification were achieved using an HPLC system (Waters® 2690 Alliance, Milford, CT, USA) integrated with a Waters 996 photodiode array detector.

##### 2.7.2.3 Operating conditions

Chromatographic separation was achieved using an Inertsil ODS C18 reversed-phase column (250 × 4.6 mm, 5 µm) at ambient temperature, with a binary mobile phase gaseous aqueous gradient of 0.1% trifluoroacetic acid in water, pH 3.5 (A), and methanol (B). The elution profile was programmed as follows: initial conditions 95% A/5% B (0 min–5 min), linear gradient to 20% A/80% B (5 min–35 min), isocratic hold (35 min–60 min), and linear return to initial conditions (60 min–70 min), followed by column re-equilibration (70 min–75 min). The analysis used a flow rate of 1.0 mL/min, a 10 µL injection volume, and UV detection at 280 nm.

##### 2.7.2.4 Sample preparation

For HPLC analysis, a 100-mg sample of the extract was precisely weighed and dissolved in methanol. The solution was sonicated for 15 min, and a 10-µL aliquot of the resulting 200 mg/mL solution was passed through a 0.22-µm nylon syringe filter prior to injection into the HPLC system.

##### 2.7.2.5 Validation analysis

The HPLC-DAD assay was validated for quantification according to ICH guidelines. The linearity was investigated by injecting seven different concentrations (5 μg/mL–200 μg/mL) of chlorogenic acid (the reference standard). For each concentration, the measurements were performed in triplicate, and the mean and the standard deviation were calculated. Then, the regression was analyzed, and the correlation coefficient *R* ^2^ was obtained. Method sensitivity was assessed through the calculation of the limit of quantification (LOQ) and limit of detection (LOD) based on the following formula: $\text{LOQ}=3\;\frac{\sigma }{S},$

$$\text{LOQ}=10\;\frac{\sigma }{S},$$

where σ = SD of the response and S = the slope of the calibration curve.

The HPLC system’s precision was confirmed by triplicate 10-µL injections of chlorogenic acid and apigenin standards, with low %RSD values confirming system suitability. Method precision was similarly validated by triplicate analysis of *D. costus* defatted extract, where acceptable %RSD values confirmed reproducibility. Additionally, solution stability was evaluated under different storage conditions, including room temperature (25 °C for 48 h) and refrigerated (4 °C for 10 days) preservation, to assess the analytical integrity over time.

### 2.8 Body weight gain (BWG)

Body weight gain was measured weekly. Upon conclusion of the experiment, abdominal and lumbar fats were excised from all rats and weighed. Relative fat weight was calculated as (fat weight/final body weight) × 100.

### 2.9 Sampling

Following an overnight fast, rats were anesthetized with isoflurane (Zhou et al., 2006), and blood was collected via retro-orbital puncture. Samples were incubated in plain vacutainers for 30 min at room temperature to permit clotting, after which centrifugation was carried out at 3,000 × g for 15 min at a temperature of 4 °C. The resultant serum fraction was aliquoted and cryopreserved at −80 °C for subsequent biochemical assays. Post-euthanasia procedures included surgical isolation of the left femoral bone, with meticulous removal of periosteal and soft tissue attachments using sterile surgical instruments, followed by physiological saline irrigation to prepare the osseous specimen for further analysis. Additionally, abdominal/lumbar adipose tissue, uterus, and liver were surgically removed, weighed, and processed for downstream examination.

### 2.10 Biochemical analysis

Serum levels of total cholesterol (TC) (Cat 11539), triglycerides (TG) (Cat 11828), high-density lipoprotein cholesterol (HDL-C) (Cat 11523), glucose (Cat 11803), and total protein (Cat 11572) were determined using standard colorimetric kits (BioSystems, Barcelona, Spain). A commercial ELISA kit (Thermo Fisher Scientific, Cat# ERINS, Invitrogen, USA) was used to measure insulin according to Zhang et al. (2020). HOMA-IR Calculation: insulin resistance was evaluated by calculating the HOMA-IR index using the standard formula: [fasting insulin (µIU/mL) × fasting glucose (mmol/L)]/22.5. Alternatively, insulin levels were also determined via a luminescence-based immunoassay (Luminex™) using kits from Linco Research™ (Millipore™) with a detection limit of 28 pg/mL. Albumin was measured using a colorimetric assay kit (Cat ab235628, abcam, UK) according to Alsagaff et al. (2022). Total calcium was estimated according to Abdelrazek et al. (2019) using enzymatic calorimetric kits (Biodiagnostic, Egypt). Ionized calcium levels (mg/dL) were calculated as following:

Ionized calcium = 0.9 + 0.55 × Y – 0.3 × Z, where Y is the total Ca (mg/dL) and Z is albumin (mg/dL) according to Temizkan et al. (2015). A specific rat ELISA kit was used for the determination of serum resistin levels (Rat RETN/Resistin (Cat: LS-F35166; Absolute, Biotech, China). Specific rat ELISA kits were used for the determination of serum adiponectin and leptin (Cat: MBS8244709, MyBioSource, USA; Cat: CSB-E07433r, CUSABIO, China), respectively. A commercial colorimetric assay kit (LDN, Germany) was used to determine the serum total antioxidant capacity (TAC) in accordance with the manufacturer’s protocol.

### 2.11 Histopathology and immunohistochemistry (IHC) preparation

The collected tissue samples (adipose tissue, uterus, femur, and liver) were fixed in 10% neutral buffered formalin for 48 h. Femurs underwent decalcification in 10% EDTA solution, with daily solution changes for 6 weeks. All tissue specimens underwent standardized histological processing through a graded ethanol dehydration series (70%, 80%, 95%, and 100% concentrations), followed by xylene clearing and paraffin embedding at 58 °C–60 °C. Tissue sections (5 μm) were serially cut on a Leica RM2235 precision rotary microtome. These sections were then mounted onto positively charged slides for subsequent histological and immunohistochemical staining. Sections of examined tissues were stained with hematoxylin and eosin according to standard protocols for general histological assessment (Grosset et al., 2019). Liver lesions were evaluated semi-quantitatively in six animals per group. Three sections per animal and five non-overlapping fields per section were assessed by an examiner blinded to group allocation. Hepatic steatosis, hepatocellular necrosis, inflammatory-cell infiltration, and sinusoidal or vascular congestion were each scored from 0 to 4 according to the proportion of affected tissue: 0, absent; 1, mild (<10%); 2, moderate (10%–30%); 3, marked (31%–50%); and 4, severe (>50%). Field-level scores were averaged within each animal, and the animal was considered the experimental unit.

Immunohistochemical detection of ER-α in uterine tissue and tumor necrosis factor alpha (TNF-α) in white adipose tissue was performed according to previously described protocols (Mann et al., 2001; Álvares et al., 2018)*.* Formalin-fixed, paraffin-embedded tissue sections of 5-µm thickness were mounted on positively charged slides, deparaffinized, and rehydrated through graded ethanol concentrations. For TNF-α immunostaining, heat-mediated antigen retrieval was performed using citrate buffer at pH 6, in accordance with the manufacturer’s recommendation. Endogenous peroxidase activity was quenched by incubation with 3% hydrogen peroxide for 15 min at room temperature, followed by blocking of non-specific binding with 5% normal goat serum for 15 min. The sections were incubated overnight at 4 °C in a humidified chamber with an anti-ER-α mouse monoclonal antibody, clone F-10, catalog number sc-8002 (Santa Cruz Biotechnology), at a dilution of 1:50 or an anti-TNF-α mouse monoclonal antibody, clone TNFA/1172, catalog number ab220210 (Abcam), at a dilution of 1:100. The manufacturer recommends an IHC-P working concentration of 2 μg/mL–4 μg/mL. Immunoreactivity was visualized using 3,3′-diaminobenzidine as the chromogen, followed by counterstaining with Mayer’s hematoxylin. Appropriate positive-control sections known to express ER-α or TNF-α were included to confirm successful immunostaining. Negative-control sections were processed in parallel using the same staining procedure, except that the primary antibody was omitted. No specific DAB immunostaining was detected in the negative-control sections. For quantitative image analysis, six randomly selected animals per group were evaluated, with three sections examined per animal. Digital images were analyzed using ImageJ software, version 1.36 (National Institutes of Health, Bethesda, MD, USA), by an investigator blinded to the experimental-group identity. ER-α and TNF-α immunoreactivity was quantified as the percentage of immune-positive area relative to the total analyzed tissue area. Measurements obtained from all examined fields and sections belonging to the same animal were averaged to generate one animal-level value. Accordingly, the individual animal was considered the experimental unit.

#### 2.11.1 Quantitative histomorphometry analyses

Quantitative histomorphometric and immunohistochemical analyses were performed in six animals per experimental group. Three sections were evaluated per animal, and five non-overlapping microscopic fields were examined per section. Fields were selected using a systematic sampling procedure that covered the tissue section while avoiding tissue folds, processing artifacts, and incomplete tissue profiles.

Digital images were analyzed using ImageJ software, version 1.36 (National Institutes of Health, Bethesda, MD, USA). Images were spatially calibrated using stage-micrometer calibration/calibrated image metadata. All measurements were performed using coded images by an investigator blinded to experimental-group identity.

Uterine histomorphometry included endometrial, myometrial, and perimetrial thicknesses and uterine-gland density. Layer thickness was measured perpendicular to the tissue surface at five locations in each section. Uterine glands were counted within the analyzed endometrial area and expressed as glands/mm^2^.

The mean adipocyte diameter was measured across intact adipocyte profiles with clearly defined cell boundaries. Tangentially sectioned, ruptured or incompletely visible cells were excluded. At least 50 adipocytes distributed across the evaluated fields were measured for each animal. Adipocytes area was also determined.

The mean trabecular thickness and trabecular area fraction were measured in femur tissue. The trabecular area fraction was calculated as the trabecular-bone area divided by the total analyzed tissue area and expressed as a percentage. In addition, the trabecular number, trabecular separation, and osteoblast/osteoclast surfaces were also determined. Measurements from all fields and sections belonging to the same animal were averaged to obtain one animal-level value. The individual animal was considered the experimental unit.

### 2.12 Statistical analysis

The individual animal was considered the experimental unit for all *in vivo* analyses. Measurements obtained from multiple sections, microscopic fields, or technical replicates from the same animal were averaged to generate one animal-level value before statistical analysis. The exact number of animals included in each analysis is stated in the corresponding table or figure legend.

Animal data are presented as the mean ± standard error, together with 95% confidence intervals where applicable. Results of the phytochemical and chemical antioxidant assays are presented as the mean ± SD of three independent analytical determinations. Statistical analyses were performed using IBM SPSS Statistics, version 20.0 (IBM Corp., Armonk, NY, USA).

Distributional assumptions were assessed by visual inspection of histograms and Q–Q plots and using the Shapiro–Wilk test. Homogeneity of variance was evaluated using Levene’s test. For continuous outcomes satisfying normality and variance-homogeneity assumptions, differences among the sham, OVX, and OVX/*D* groups were analyzed using one-way analysis of variance, followed by Tukey’s honestly significant difference test for adjusted pairwise comparisons.

When the assumption of equal variances was not satisfied, Welch’s one-way analysis of variance followed by the Games–Howell *post hoc* test was used. Outcomes showing substantial departures from normality were analyzed using the Kruskal–Wallis test, followed, when appropriate, by Dunn’s pairwise-comparison test with Holm adjustment for multiple comparisons. Ordinal lesion scores, where applicable, were analyzed using the Kruskal–Wallis and adjusted Dunn procedures.

For parametric analyses, the overall *F* statistic, numerator and denominator degrees of freedom, exact two-sided *p-*value, eta-squared effect size (η^2^), and 95% confidence intervals were reported. For Kruskal–Wallis analyses, the *H* statistic, degrees of freedom, exact *p-*value, and epsilon-squared effect size (ε^2^) were reported. Adjusted exact *p-*values and 95% confidence intervals for pairwise mean or median differences were reported where applicable. Statistical significance was defined as a two-sided *p* < 0.05; values below 0.001 were reported as *p* < 0.001.

Semi-quantitative histopathological lesion scores (graded on an ordinal scale of 0–4 for individual parameters and 0–16 for total scores) were analyzed using the non-parametric Kruskal–Wallis test followed by Dunn’s post hoc test with Holm’s adjustment for multiple pairwise group comparisons. For descriptive presentation and consistency across outcome measures, ordinal score data are presented as mean ± standard error (SE) alongside median and interquartile range (IQR). Statistical significance was established at *p* < 0.05.

Correlation analyses were not performed because individual biochemical, histomorphometric, and immunohistochemical measurements could not be unequivocally matched across all outcomes for the same animals. Correlations were not calculated from group means, and data from the three experimental groups were not pooled for unadjusted correlation analysis because such an approach could produce associations driven mainly by treatment-group differences.

## 3 Results

### 3.1 Total phenolic, flavonoids, and terpenoids contents

As shown in Table 1, the phytochemical analysis of *D. costus* crude extract revealed the following composition: the Folin–Ciocalteu assay measured a TPC of 70.6 ± 4.8 mg gallic acid equivalents (GAE) per gram extract, while aluminum chloride (AlCl_3_) spectrophotometric analysis determined a TFC of 29.8 ± 2.6 mg quercetin equivalents (QE) per gram. Additionally, the Salkowski method quantified the TTC as 10.3 ± 2.4 mg linoleic acid equivalents per gram of extract.

**TABLE 1:** Total phenolic, flavonoids, and terpenoids contents of *Dolomiaea costus (*Syn*. Saussurea costus)* roots extract.

Sample	TPC (mg GAE/g)	TFC (mgQE/g)	TTeC (mg linol/g)
*D. costus* crude extract	70.6. ± 4.8	29.8 ± 2.6	10.3 ± 2.4

Values are expressed as the mean ± standard deviation (SD) of three independent measurements. TPC, total phenolic content; TFC, total flavonoids content; TTeC, total terpenoids content; GAE, gallic acid equivalents.

### 3.2 Chemical antioxidant activity of *Dolomiaea costus* root extract

The extract demonstrated measurable antioxidant activity across the four complementary chemical assays (Table 2). In the DPPH assay, the extract showed a chemical IC_50_ of 213.78 ± 3.95 μg/mL, compared with 10.64 ± 0.82 μg/mL for ascorbic acid. The ABTS radical-cation scavenging chemical IC_50_ was 248.74 ± 7.42 μg/mL, compared with 141.98 ± 3.46 μg/mL for BHT. The extract exhibited a FRAP value of 1.85 ± 0.39 mmol Fe^2+^ equivalents/g and a phosphomolybdenum reducing capacity of 34.86 ± 2.02 mg GAE/g. Collectively, these findings indicate moderate, assay-dependent chemical radical-scavenging and reducing activity relative to the reference antioxidants.

**TABLE 2:** Chemical radical-scavenging and reducing capacities of the *Dolomiaea costus (Syn. Saussurea costus)* roots extract measured using DPPH, ABTS, FRAP, and phosphomolybdenum assays.

Sample	DPPH (IC_50_ in µg/mL)	ABTS (IC_50_ in µg/mL)	FRAP (mMol Fe^+2^/g)	Phosphomolybdenum reducing capacity (mg GAE/g)
*D. costus* crude extract	213.78 ± 3.95	248.74 ± 7.42	1.85 ± 0.39	34.86 ± 2.02
BHT	NT	141.98 ± 3.46	2.86 ± 0.38	79.31 ± 3.95
Ascorbic acid	10.64 ± 0.82	NT	7.41 ± 0.57	72.68 ± 3.74

Data are expressed as the mean ± SD (n = 3). NT, not tested; DPPH, 2,2-diphenyl-1-picrylhydrazyl; FRAP, ferric reducing antioxidant power; TAC, total antioxidant capacity; Fe^2+^, iron; GAE, gallic acid equivalents.

### 3.3 Chemical profiling of *Dolomiaea costus* extract

#### 3.3.1 Gas chromatography–mass spectrometry (GC–MS) of *Dolomiaea costus*

GC–MS analysis of the unsaponifiable and saponifiable fractions produced a series of peaks that were provisionally annotated by comparison with the NIST17 s.lib. The proposed assignments, retention data, relative peak areas, and library-match scores (SI) are presented in Tables 3 and 4. The identified compounds were filtered to include only compounds with SI ≥ 80 based on NIST guidelines (SI > 90 is excellent match; SI 90–80 is good match (Kirchkeszner et al., 2022). Because authentic GC–MS reference standards were not analyzed, the reported compound names represent putative annotations rather than unequivocal structural identifications.

**TABLE 3:** Results of GC–MS analysis of the unsaponifable fraction of the *n*-hexane extract of *Dolomiaea costus roots*.

Compound tentatively annotated	Rt (min)	RRt (min)	Peak area %	SI	Molecular formula	Molecular weight
3,3 Diethoxy-1,2-propanediol	14.936	0.437	0.71	88	C_7_H_16_O_4_	164.2
3-Ethyl-3-hydroxyandrostan-17-one	27.640	0.808	3.06	85	C_21_H_34_O_2_	318.5
(8Z,11Z,14Z) Heptadeca-1,8,11,14-tetraene	28.106	0.822	4.91	95	C_17_H_28_	232.4
Nopol	28.848	0.844	0.92	83	C_11_H_18_O	166.3
6-Phenyldodecane	29.485	0.862	0.9	91	C_18_H_30_	246.4
5-Phenyldodecane	29.582	0.865	0.93	86	C_18_H_30_	246.4
cis-Lanceol	29.964	0.876	6.89	80	C_15_H_24_O	220.4
γCostol	30.120	0.881	2.55	87	C_15_H_24_O	220.4
3-Phenyldodecane	30.335	0.887	0.65	87	C_18_H_30_	246.4
Dehydrosaussurea lactone	30.951	0.905	1.29	91	C_15_H_20_O_2_	232.3
2-Phenyldodecane	31.145	0.910	0.57	95	C_18_H_30_	246.4
11β,13-Dihydrodehydrocostus lactone (Mokkolactone)	33.141	0.969	4.71	93	C_15_H_20_O_2_	232.3
Costunolide (Dihydrodehydrocostus lactone)	34.075	0.997	2.58	88	C_15_H_20_O_2_	232.3
Dehydrocostus lactone	34.188	1	15.38	92	C_15_H_18_O_2_	230.3
Dihydrodehydrocostus lactone isomer	37.148	1.087	8.02	80	C_15_H_20_O_2_	232.3
*n*-Eicosane	42.693	1.249	1.09	83	C_20_H_42_	282.5
*n*-Tetratriacontane	44.324	1.296	1.61	82	C_34_H_70_	478.9
*n*-Pentatriacontane	45.889	1.342	2.01	83	C_35_H_72_	492.9
*n*-Hexatriacontane	47.404	1.387	2.00	82	C_36_H_74_	506.9
Bis (2-ethylhexyl) phthalate	47.668	1.394	1.5	84	C_24_H_38_O_4_	390.5
*n*-Tetracontane	48.857	1.429	1.80	81	C_40_H_82_	563.1
*n*-Tetratetracontane	50.264	1.470	1.68	80	C_44_H_90_	619.2
Carbonic acid, decyl octadecyl ester	54.309	1.589	1.09	71	C_29_H_58_O_3_	917.3
Strophanthidol	55.715	1.629	2.24	73	C_23_H_34_O_6_	454.8
Octatriacontyl trifluoroacetate	58.915	1.723	24.30	73	C_40_H_77_F_3_O_2_	647.0

RT, retention time; RRt, retention time relative to dehydrocostus lactone; SI, similarity index (match threshold >80%).

**TABLE 4:** Results of GC–MS analysis of the saponifiable fraction of the *n*-hexane extract of *Dolomiaea costus roots*.

Compound tentatively annotated	Rt (min)	RRt (min)	Peak area %	SI	Molecular formula	Molecular weight
Methyl-β-costate	30.440	0.826	2.69	89	C_16_H_24_O_2_	248.4
Saussurea lactone	31.021	0.841	4.54	95	C_15_H_20_O_2_	232.3
Dihydrodehydrocostus lactone	31.964	0.867	0.25	86	C_15_H_20_O_2_	232.3
Dehydrocostus lactone	33.002	0.896	9.79	86	C_15_H_18_O_2_	230.3
Dihydro-β-cyclocostunolide	33.085	0.898	0.50	89	C_15_H_22_O_2_	234.3
Palmitic acid, methyl ester	33.641	0.913	8.48	94	C_17_H_34_O_2_	270.5
Dehydrocostus lactone	34.193	0.928	3.76	93	C_15_H_18_O_2_	230.3
Palmitic acid	34.298	0.931	0.67	90	C_16_H_32_O_2_	256.4
Linoleic acid, methyl ester	36.853	1	12.19	95	C_19_H_32_O_2_	292.2
Elaidic acid, methyl ester	37.014	1.004	2.20	92	C_19_H_36_O_2_	296.5
cis-6-Octadecenoic acid, methyl ester	37.119	1.007	0.72	95	C_19_H_36_O_2_	296.4
Linoleyl acetate	37.482	1.017	0.78	83	C_18_H_34_O	266.5
Octadecanoic acid, methyl ester	37.575	1.011	0.61	92	C_19_H_38_O_2_	298.5
Oleamide	41.193	1.118	0.69	82	C_18_H_35_NO	281.5
6-Octadecenoic acid, methyl ester	45.645	1.239	0.17	80	C_19_H_34_O_2_	294.5
cis-15-Tetracosenoic acid, methyl ester (methyl nervonate)	47.184	1.280	0.29	80	C_25_H_48_O_2_	380.6

RT, retention time; RRt: retention time relative to linoleic acid, methyl ester; SI, similarity index (match threshold >80%).

The *n*-hexane fraction of *D. costus* was saponified, and the obtained saponifiable and unsaponifiable sub-fractions were analyzed by HS.GC–MS. The results are depicted in Tables 3, 4 and Figure 1. The analysis of the unsaponifable fraction (Table 3) showed that 25 compounds were putatively identified representing 93.93% of the unsaponifiable part of *D.costus*. Octatriacontyl trifluoroacetate is the major detected component, with a percentage of 24.3. Sesquiterpene lactones constituted 31.98% of the unsaponified part, among which dehydrocostus lactone predominated (15.38%). Sesquiterpenes accounted for 12.5% of *D. costus* unsaponifiable matter, where cis-lanceol (6.89%) was the major identified compound belonging to this chemical class. Hydrocarbons represented 21.28% of unsaponified components, of which (8Z,11Z,14Z)-heptadeca-1,8,11,14-tetraene constituted 4.91% of them.

**FIGURE 1:**
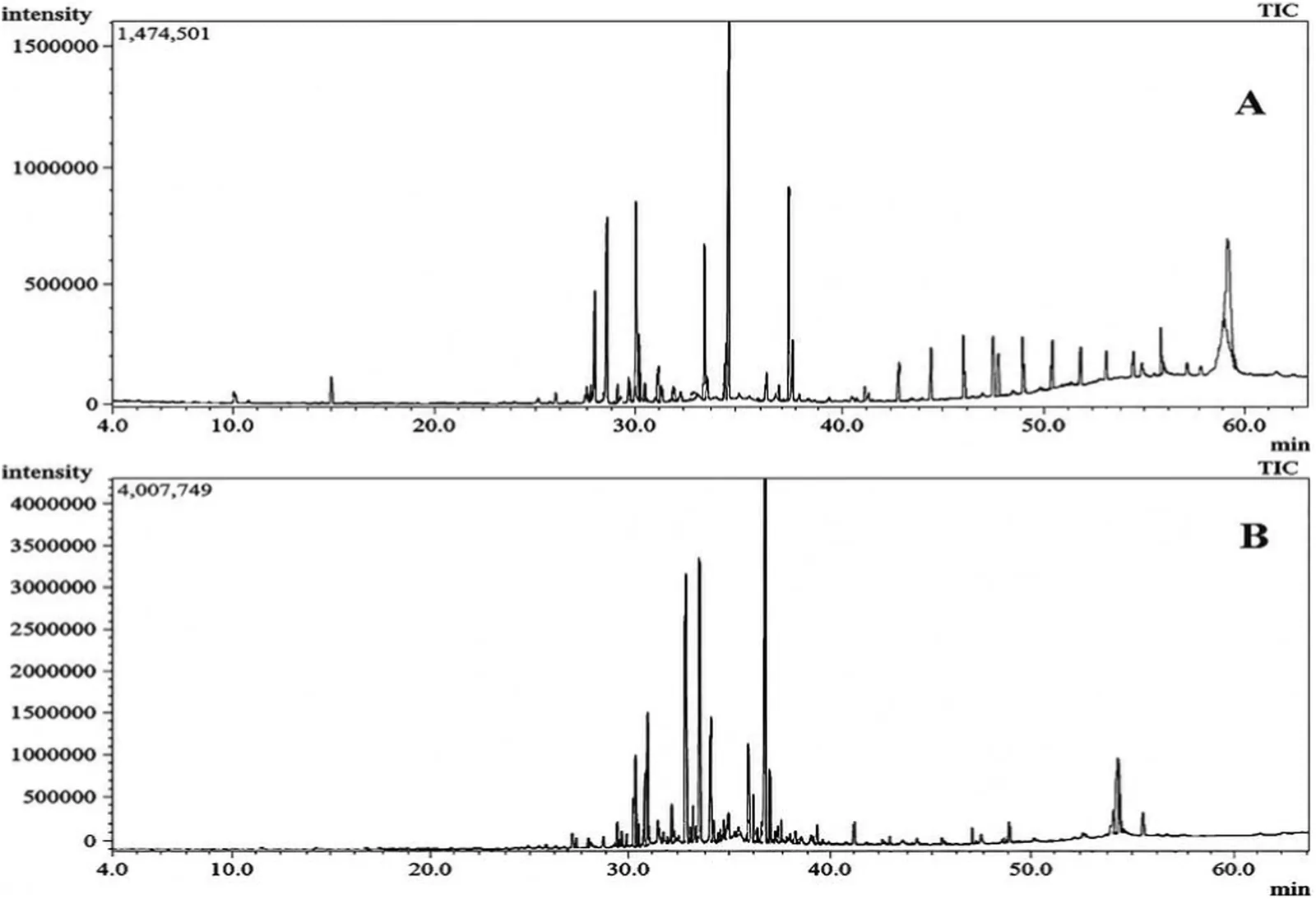
GC–MS chromatograms of the unsaponifiable fraction **(A)** and saponifiable fraction **(B)** of the *n*-hexane extract of *Dolomiaea costus (*Syn*. Saussurea costus)* roots extract.

On the other hand, analysis of the saponified portion of *D. costus* hexane fraction has led to the estimation of eight fatty acids or their esters accounting for 25.33% of the saponifiable components, where the unsaturated fatty acids and saturated fatty acids constitute 15.57% and 9.76%, respectively, of the saponifiable part of *D. costus* extract. Among the detected fatty acids, linoleic acid and palmitic acid were the major ones with relative abundances of 12.18% and 8.48%, respectively. Interestingly, several lactones, especially sesquiterpene lactones, were detected in the saponified part of *D. costus* hexane fraction, among which dehydrocostus lactone is the predominant one (9.79%). The unexpected detection of sesquiterpene lactones in this fraction could be explained based on the susceptibility of lactones due to their ring-strained nature to alkaline hydrolysis and lactone opening (Gómez-Bombarelli et al., 2013) in the saponification step of the hexane fraction, followed by re-lactonization by the action of acids (San Feliciano et al., 1986; Mulzer, 1996) in the neutralization and methylation procedures.

#### 3.3.2 HPLC-DAD analysis of defatted extract of *Dolomiaea costus* roots

HPLC-DAD analysis was utilized for chemical profiling of defatted extract of *D. costus* roots. Qualitative identification of polyphenolics in the extract was accomplished by comparing the UV spectra together with the retention time of the generated peaks from *D*. *costus* with those of 10 available phenolics that are mostly abundant in plants (four phenolic acids: caffeic, ellagic, chlorogenic, and gallic acid and six flavonoids: kaempferol, apigenin, rutin, hesperidin, quercetin, and catechin) The peak detected at 25.96 min was identified as chlorogenic acid, while the other compounds were present in trace or sub-detected amounts (Figures 2, 3). Therefore, a HPLC-DAD method was established for quantification of chlorogenic acid in *D. costus* using the external standard method. Validation parameters of the HPLC-DAD method are shown in Table 5. The method exhibited excellent linearity for chlorogenic acid, with a coefficient of determination (*R* ^2^) of 0.999. The LOD and LOQ values were suitable for quantification. The method was sensitive since the LOD and LOQ for chlorogenic acid were 0.064 μg/mL and 0.21 μg/mL, respectively. The system and method precision were assured by low percentages of RSD, which were 2.73% and 0.54%, respectively. The estimation of chlorogenic acid content in *D. costus* was achieved using the proposed method of analysis. The area under the peak was measured three times, and then the concentration was calculated by applying the regression equation.

**FIGURE 2:**
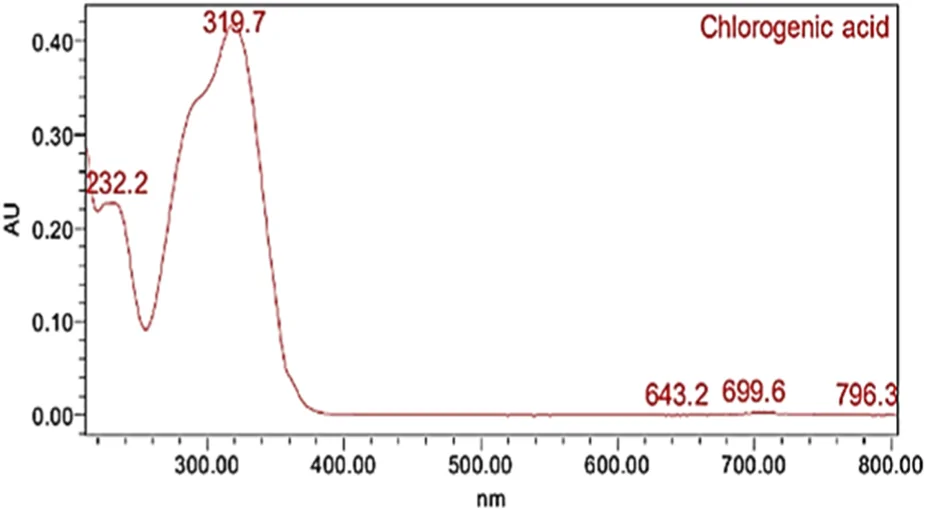
UV–VIS absorbing spectrogram of chlorogenic acid.

**FIGURE 3:**
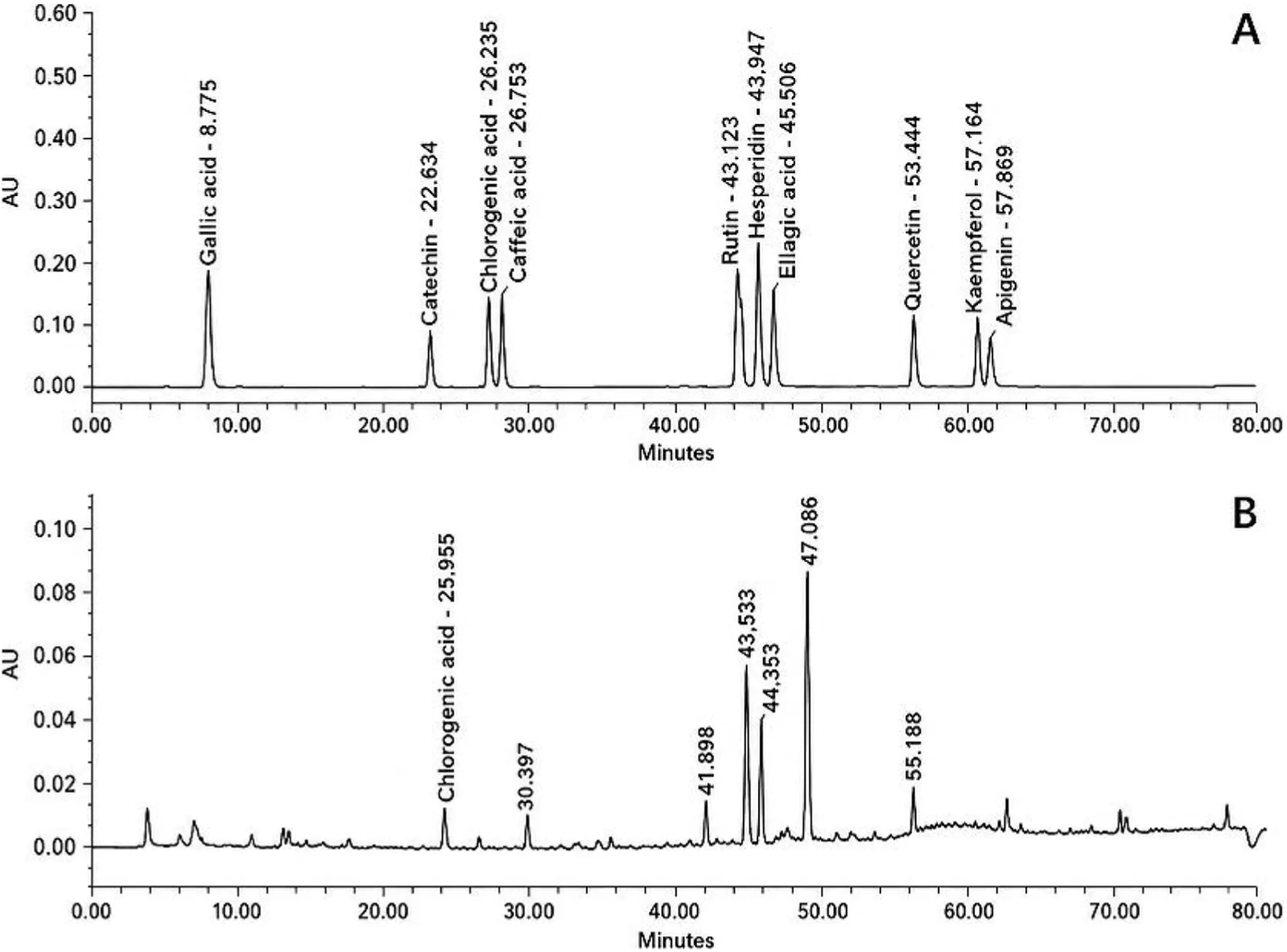
HPLC chromatograms of **(A)** reference standards and **(B)** *Dolomiaea costus* (Syn. *Saussurea costus*) roots defatted extract, monitored at λ = 280 nm.

**TABLE 5:** Validation parameters of the HPLC method for quantitative analysis of chlorogenic acid in *Dolomiaea costus* roots extract.

Validation parameter	Chlorogenic acid
Linearity range (µg/mL)	5–125
Regression equation	y = 25,263.687x + 38,473.594
Correlation coefficient (R^2^)	0.999
System precision (%RSD)	2.73
Method precision (%RSD)	0.54
Limit of detection (µg/mL)	0.064
Limit of quantification (µg/mL)	0.21
Concentration in the sample (mg/gm)	0.66±0.003

Scanned at λ = 280 nm; %RSD, relative standard deviation.

HPLC-DAD peak assignments were evaluated by comparison of the retention times and UV absorption spectra with those of the 10 authentic phenolic reference standards analyzed under the same chromatographic conditions. Chlorogenic acid was identified by the agreement of its retention time and UV spectrum with the authentic standard, which represents the first level of identification confidence according to Schymanski et al. (2015) and was quantified using external-standard calibration to be 0.66 ± 0.003 mg/g extract. The other investigated phenolic compounds were either detected at trace levels or remained below the applicable quantification limit and were, therefore not reported as quantitatively confirmed constituents.

### 3.4 Initial and final weight gain, fat weight, and relative fat weight

Table 6 shows the fat weight in addition to the relative fat weight of the sham, OXV, and OVX/*D* groups. It also shows the initial weight, final weight, and weight gain. The initial body weight did not differ among the three groups, $F\left(2,27\right)=0.365$ , *p* = 0.697, and η^2^ = 0.026, confirming comparable baseline body weights.

**TABLE 6:** Results of the impact of *Dolomiaea costus (*Syn*. Saussurea costus)* on weight gain and adipose tissue metrics (absolute mass and relative mass) in treated female rats.

Parameters	Sham	OVX	OVX/*D*	Overall ANOVA	Exact *p*	η^2^
Initial weight (g)	110.7 ± 1.5^a^	109.7 ± 1.1^a^	109.3 ± 0.9^a^	*F* (2,27) = 0.365	0.697	0.026
Final body weight (g)	228.3 ± 7.3^b^	297.7 ± 7.0^b^	246.3 ± 9.8^b^	*F* (2,27) = 19.620	<0.001	0.592
Weight gain (g)	117.7 ± 6.3^b^	188.0 ± 7.1^b^	137.0 ± 10.5^b^	*F* (2,27) = 19.754	<0.001	0.594
Fat weight (g)	9.7 ± 0.5^b^	15.9 ± 1.3^a^	7.9 ± 0.4^b^	*F* (2,27) = 25.162	<0.001	0.651
Relative fat weight	4.3 ± 0.3a^b^	5.4 ± 0.5^a^	3.2 ± 0.2^b^	*F* (2,27) = 9.553	<0.001	0.414

Data are presented as the mean ± SEM, with *n* = 10 independent animals per group. The individual animal was considered the experimental unit. Group differences were evaluated using one-way ANOVA, followed by Tukey’s HSD test. The overall *F* statistic, degrees of freedom, exact two-sided *p-*value, and eta-squared effect size (η^2^) are presented for each outcome. Different superscript letters indicate significant Tukey-adjusted pairwise differences at *p* < 0.05. OVX, ovariectomized group; OVX/D, ovariectomized group administered 300 mg/kg body weight of *Dolomiaea costus* (Syn. *Saussurea costus*) roots extract.

In contrast to the sham and OVX/*D* groups, the OVX group demonstrated a statistically significant increase in the final body weight, weight gain, and fat weight. No notable differences were observed in the earlier parameters. The final body weight differed significantly among groups, $F\left(2,27\right)=19.620$ , *p* < 0.001, and η^2^ = 0.592. Tukey’s test showed that the final body weight was higher in the OVX group than in both the sham and OVX/*D* groups. However, the relative fat weight was significantly higher in the OVX/*D* group than in the OVX groups alone.

### 3.5 Biochemical analysis

Table 7 displayed the impact of *D. costus* on the biochemical parameters of animals that had their ovaries removed. In terms of lipid profiles, the OVX group showed significantly higher serum levels of TG and TC versus sham and OVX/*D* groups. Compared to other groups, the OVX group experienced a significant decrease in HDL-C levels.

**TABLE 7:** Effect of *Dolomiaea costus* (Syn. *Saussurea costus*) roots extract on biochemical parameters in treated female rats.

Parameter	Sham	OVX	OVX/*D*
Total cholesterol (mg/dL)	50.2 ± 2.0^b^	68.5 ± 3.2^a^	54.4 ± 1.7^b^
Triglycerides (mg/dL)	82.7 ± 2.3^c^	115.8 ± 2.2^a^	95.8 ± 3.0^b^
HDL-C (mg/dL)	16.1 ± 0.3^a^	10.4 ± 0.2^c^	14.3 ± 0.2^b^
Insulin (ng/mL)	1.2 ± 0.0^a^	1.1 ± 0.0^b^	1.2 ± 0.0^a^
Glucose (mg/dL)	82.3 ± 1.8^c^	113.7 ± 2.0^a^	91.3 ± 1.1^b^
HOMA-IR	0.24 ± 0.0^c^	0.33 ± 0.0^a^	0.27 ± 0.0^b^
Albumin (g/dL)	4.4 ± 0.0^a^	4.2 ± 0.0^c^	4.3 ± 0.0^b^
Total protein (g/dL)	6.1 ± 0.0^a^	5.8 ± 0.0^c^	5.9 ± 0.0^b^
Calcium (mg/dL)	8.1 ± 0.0^a^	7.0 ± 0.0^c^	7.5 ± 0.0^b^
Ionized calcium (µmol/L)	740.0 ± 0.3^a^	636.8 ± 1.8^c^	682.1 ± 2.3^b^
Resistin (ng/mL)	7.3 ± 0.0^c^	11.6 ± 0.0^a^	8.9 ± 0.0^b^
Adiponectin (µg/mL)	8.7 ± 0.0^a^	6.4 ± 0.0^c^	7.6 ± 0.0^b^
Leptin (ng/mL)	2.6 ± 0.0^c^	3.8 ± 0.0^a^	3.1 ± 0.0^b^
TAC (nmol/mL)	0.8 ± 0.0^a^	0.3 ± 0.0^b^	0.7 ± 0.0^a^

Data are presented as the mean ± SEM, with *n* = 10 independent animals per group. Group differences were evaluated using one-way ANOVA, followed by Tukey’s HSD test. The individual animal was considered the experimental unit. The overall test statistic, degrees of freedom, exact two-sided *p-*value, and η^2^ are reported for each outcome. Different superscript letters indicate significant Tukey-adjusted pairwise differences at *p* < 0.05. OVX, ovariectomized group; OVX/D, ovariectomized group administered 300 mg/kg body weight *Dolomiaea costus* (Syn. *Saussurea costus*) roots extract; HDL, high-density lipoprotein cholesterol; HOMA-IR, homeostatic model assessment of insulin resistance; TAC, total antioxidant capacity.

In terms of metabolic profiles, the OVX group showed a significant decrease in serum insulin and upregulation of glucose when compared to the sham group and the OVX/*D* group. The HOMA-IR levels were the highest in the OVX group compared to the sham and OVX/*D* groups. Compared to other groups, the OVX group showed significantly low levels of albumin, total protein, total calcium, and ionized calcium. In addition, there was a noticeable increase in the prior parameters in the sham group when compared to those in the OVX/*D* group.

Compared to other groups, the OVX group showed significantly higher levels of resistin and leptin. In addition, the adiponectin and TAC parameters showed a notable (*p* < 0.05) increase in the OVX/*D* group when compared to the OVX group.

Significant group effects were detected for TC, *F* (2,27) = 16.093, *p* < 0.001, and η^2^ = 0.544; TG, *F* (2,27) = 43.576, *p* < 0.001, and η^2^ = 0.763; HDL-C, *F* (2,27) = 149.824, *p* < 0.001, and η^2^ = 0.917; glucose, *F* (2,27) = 92.824, *p* < 0.001, and η^2^ = 0.873; and ionized calcium, *F* (2,27) = 931.249, *p* < 0.001, and η^2^ = 0.986. Tukey-adjusted comparisons indicated that untreated OVX animals had higher TC, TG, and glucose and lower HDL-C and ionized calcium than sham-operated animals. Treatment with *D. costus* was associated with values intermediate between or closer to the sham group, depending on the outcome.

### 3.6 Histopathological, immunohistochemical, and morphometric results

#### 3.6.1 Histopathological, immunohistochemical, and morphometric results of uterus

Histological examination of uterine tissues demonstrated distinct anatomical organization in sham rats, with three clearly identifiable layers radiating outward from the central lumen: (1) the innermost endometrium, (2) middle myometrium, and (3) outer perimetrium. The sham-operated group exhibited normal uterine histoarchitecture, with all three tunics maintaining healthy morphological characteristics and proper structural integrity. The endometrial mucosa appeared folded and was surfaced by a single layer of columnar epithelial cells. Beneath this, the lamina propria-submucosa consisted of loose connective tissue housing blood vessels of various calibers and uterine tubular glands. The myometrium was organized into an inner circular and an outer longitudinal layer of smooth muscle cells, divided by the stratum vasculare—a region rich in arterial and venous vessels. The outermost layer, the perimetrium, was formed of connective tissue overlaid by a simple squamous mesothelial epithelium (Figure 4).

**FIGURE 4:**
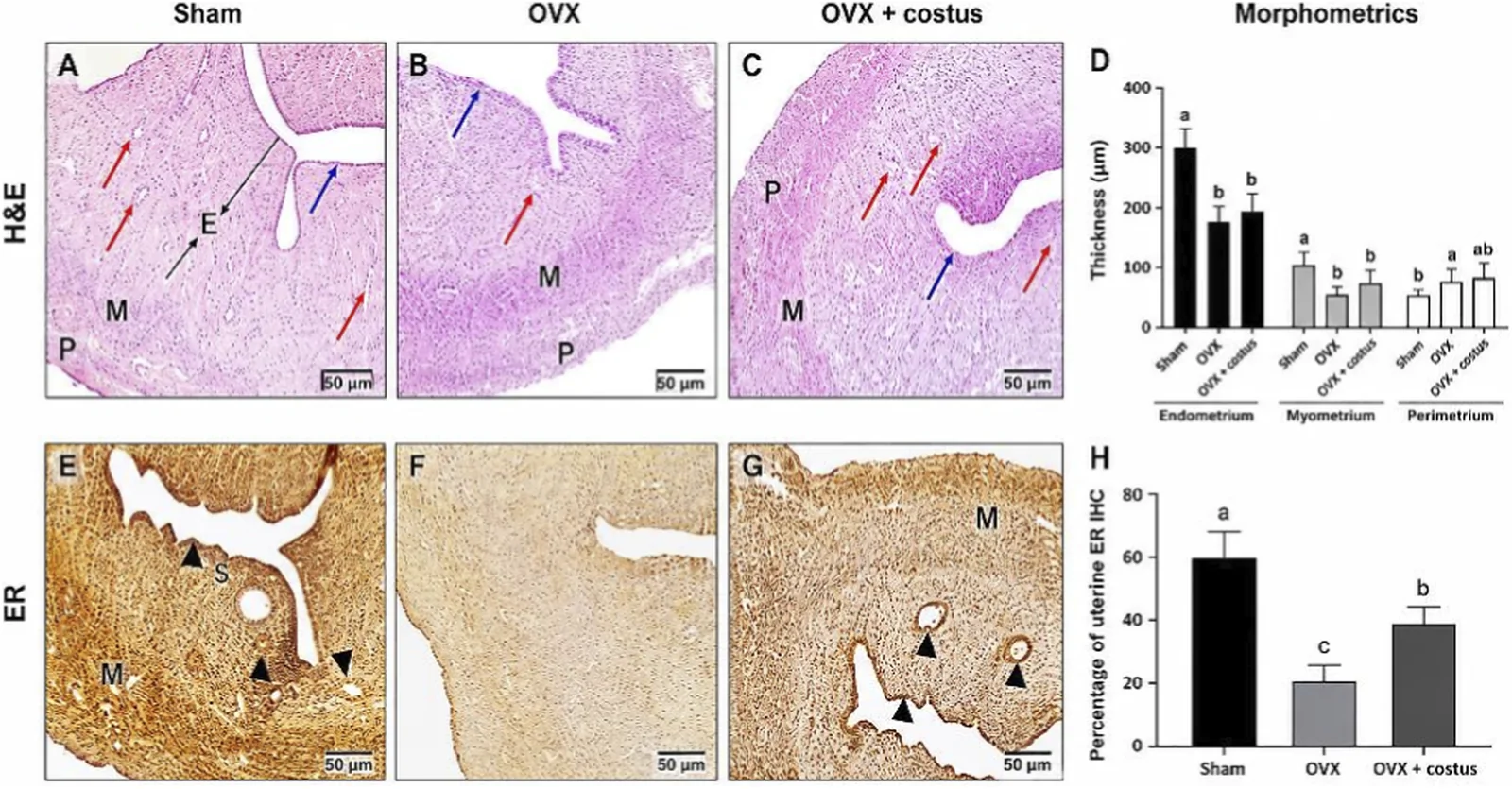
Photomicrographs of H&E- and estrogen receptor alpha (ER- α)-stained uterine sections of **(A,E)** sham, **(B,F)** OVX, and **(C,G)** *D. costus* groups. Endometrium (E), uterine epithelium (blue arrows), uterine glands (red arrows), myometrium (M), and perimetrium (P). Concerning ER immunostaining, a strong reaction was observed in luminal and glandular epithelia (arrowheads), the superficial propria-submucosa (S), and the myometrium (M) of the sham group. ER immunolabelling became faint in uterine epithelia, stroma, and myometrium of the OVX group, which was again seen in luminal, glandular epithelia, and myometrium of the OVX/*D* group (100 X), Morphometric analysis **(D,H)** showing the percentage of uterine ER-positive cells across treatment groups. Ovariectomy (OVX) significantly reduced ER-α expression compared to that in the sham group, while *Dolomiaea costus* treatment partially restored the ER-α levels.

The histopathological changes in the uteri of OVX animals were examined in comparison to that of sham. The main uterine changes were in the form of a decrease in luminal folding, reduction in the number of endometrial glands, some cellular vacuolization, very evident vascularization, and increased deposition of collagen fibers in both endo- and perimetrium. Moreover, the uterine epithelial cells were composed of a single layer of cuboidal cells. Histological examination revealed significant inflammatory cell infiltration within the lamina propria-submucosal layer directly beneath the epithelial lining, along with prominent vascular proliferation in the stratum vascular region (Figure 4). In the OVX/D group, histological improvements restored near-normal uterine architecture comparable to that in sham controls. The treatment preserved the simple columnar epithelial lining of the uterine lumen, increased endometrial gland density, and reduced stromal fibrosis (Figure 4).

Immunohistochemical analysis of ER-α expression (Figure 4) revealed distinct patterns across groups: Sham-operated rats exhibited strong ER immunoreactivity throughout the uterine tissues, including luminal/glandular epithelium, subepithelial stroma, vascular endothelium, myometrium, and perimetrium. OVX rats showed markedly diminished ER positivity, with only scattered immunoreactive cells in the vascular endothelium, myometrium, and perimetrium. In contrast, *D. costus*-treated animals demonstrated restored ER expression, characterized by prominent staining in endometrial epithelial and glandular cells, along with positive reactivity in myometrial smooth muscle fibers and perimetrial connective tissue.

There was a significant decline in endometrial and myometrium thickness, along with a significant decrease in the number uterine glands of the OVX group compared to that in the sham group. Additionally, the *D. costus* group was noted to have significantly increased endometrial and myometrium thickness and increased number of uterine glands in comparison with the OVX group. Between both the sham and *D. costus* groups, no significant differences were observed in endometrial and myometrial thickness and the number of uterine glands. On the other hand, morphometric values of perimetrium were observed to be significantly increased in the OVX group than in the sham one.

#### 3.6.2 Histological evaluation of the white adipose tissue, femur bone, and liver

Histological evaluation of the white adipose tissues of all experimental animals using H&E staining and TNF-α immunolabelling are illustrated in Figure 5. The adipose tissue of the sham group consisted mainly of adipocytes and occasional fine connective tissue elements that were found in between. The blood vessels were normal sized. They were intermittently observed within the connective tissue elements. Normal structure appearance was observed. Adipocytes exhibited smooth contours, uniform sizes, and peripheral, flattened, uni-vacuolar nuclei. Compared to that in the sham group, adipocytes in the OVX group displayed a more deformed morphology.

**FIGURE 5:**
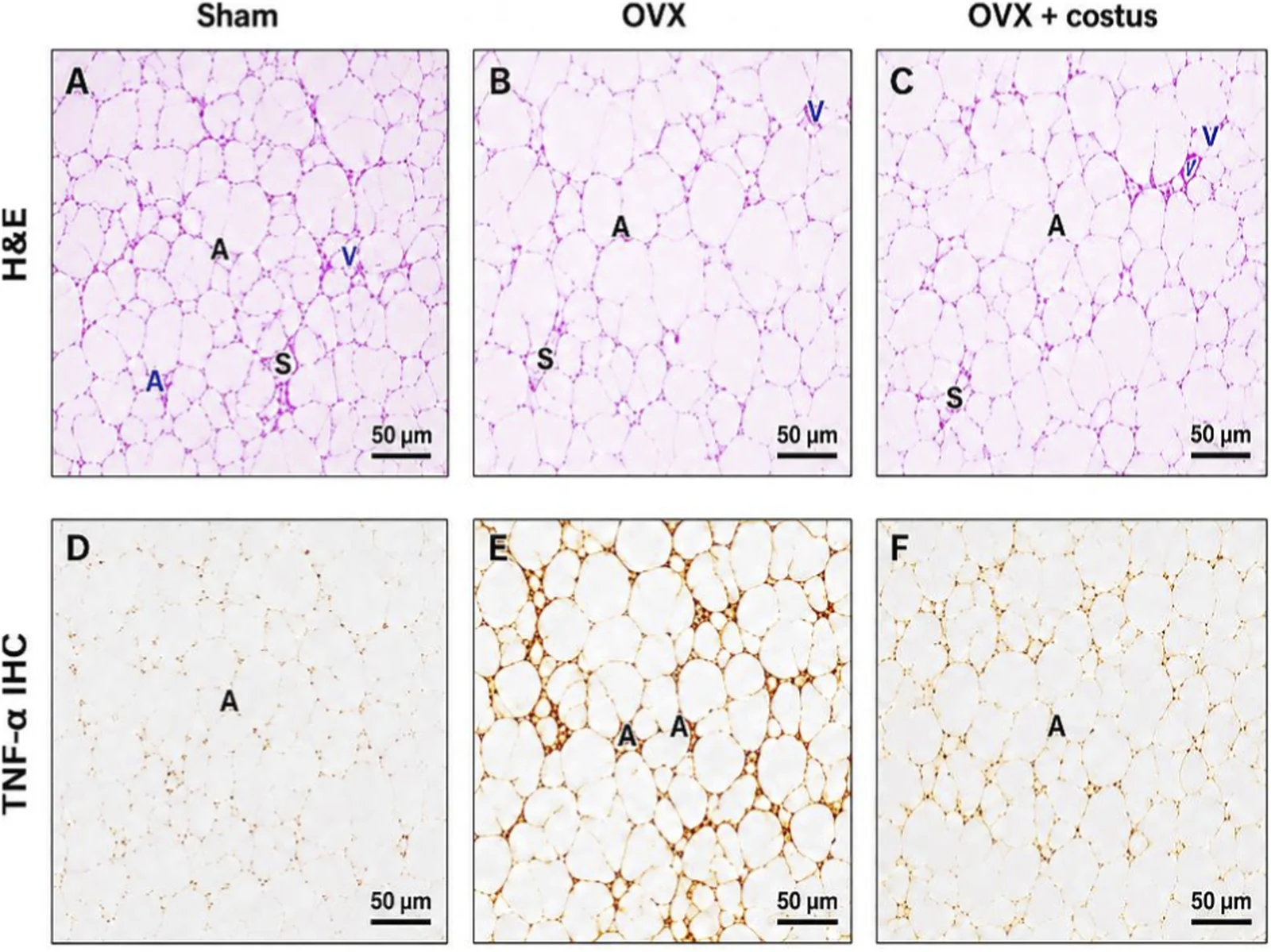
Effects of ovariectomy and *Dolomiaea costus (*Syn*. Saussurea costus)* extract on white adipose-tissue morphology and tumor necrosis factor-alpha (TNF-α) immunoreactivity. Representative photomicrographs of hematoxylin and eosin-stained white adipose tissue from the sham-operated **(A)**, ovariectomized (OVX) and **(B)**, and OVX/*D* groups **(C)**. TNF-α immunohistochemical staining is shown for the sham **(D)**, OVX **(E)**, and OVX/*D* groups **(F)**, with positive immunoreactivity visualized as brown DAB staining.

The histological examination revealed occasionally prominent, enlarged blood vessels and adipocyte hypertrophy. On the other hand, microscopic examinations of adipose tissues OVX/D group exhibited similar architecture as that of the sham one. The sham group showed weak immunoreactivity for TNF-α within connective tissue elements and blood vessels surrounding adipocytes. On the other hand, OVX group revealed strong TNF-α +ve CT cells. Compared to the OVX group, the OVX/*D* group showed weak to moderate TNF-α immuno-expression within connective tissue elements and blood vessels surrounding the adipocytes.

Transverse femoral sections (Figure 6) revealed distinct histological profiles: Sham rats (Figure 6A) exhibited thick, interconnected trabeculae with homogeneous eosinophilic matrix, enclosing marrow cavities containing hematopoietic tissue, scattered adipocytes, and blood sinusoids. In contrast, OVX rats (Figure 6B) display thin, fragmented trabeculae and expanded marrow cavities dominated by adipocytes. The OVX/*D* group (Figure 6C) showed near-normal architecture characterized by thick, branching/anastomosing trabeculae with preserved eosinophilic matrix and cellular marrow containing both hematopoietic elements and adipocytes.

**FIGURE 6:**
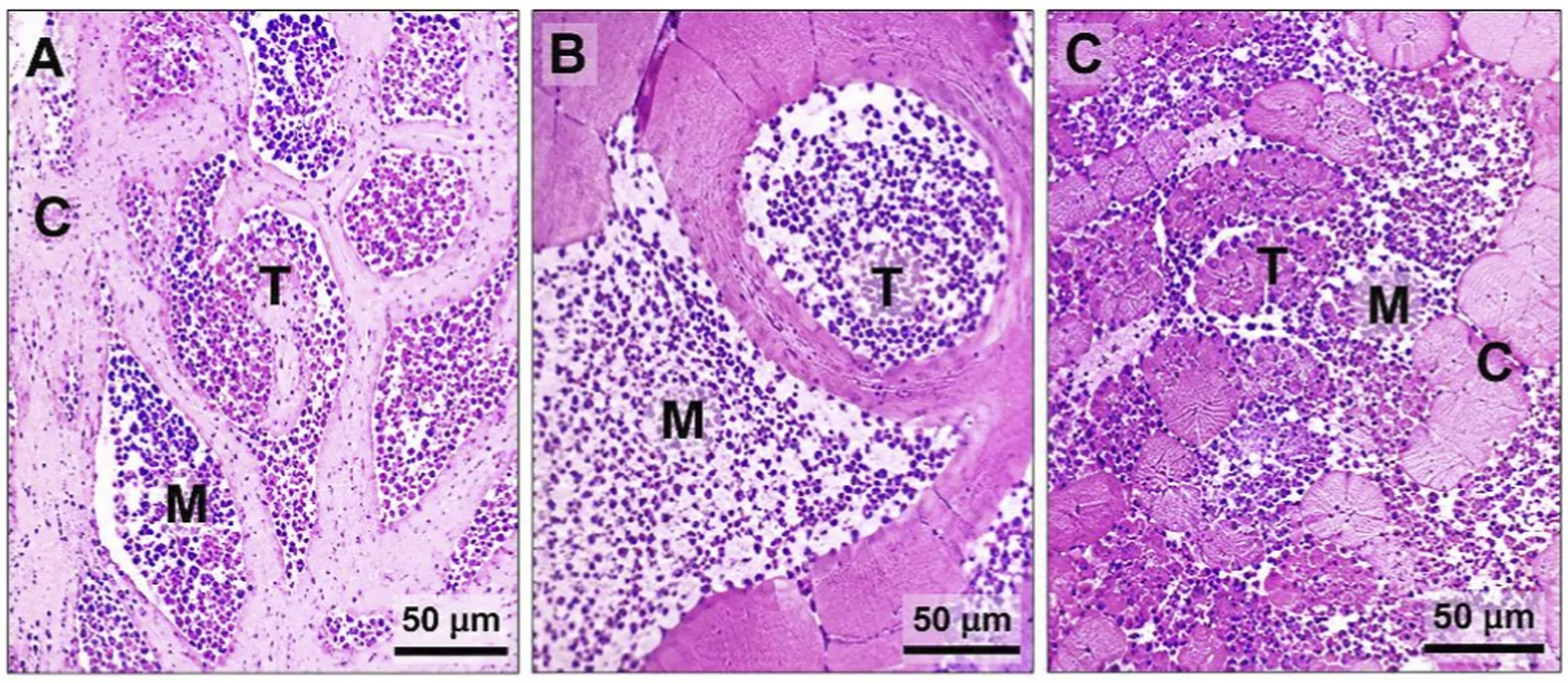
Photomicrographs of H&E-stained femoral sections revealed distinct histological profiles: **(A)** Sham rats exhibited cartilaginous tissue (C) surrounding thick trabeculae (T) with homogeneous eosinophilic matrix, enclosing hematopoietic marrow cavities (M) containing scattered adipocytes and blood sinusoids. **(B)** Conversely, OVX specimens displayed fragmented thin trabeculae (T) within cartilaginous bone, with expanded marrow cavities (M) dominated by adipocytes. **(C)** The OVX/*D* group demonstrated near-normal restoration, featuring thick anastomosing trabeculae (T) with preserved eosinophilic matrix and cellular marrow (M) containing both sinusoids and adipocytes, all maintained within cartilaginous tissue (C) (100X).

Transverse sections of the liver (Figure 7) from the sham group revealed normal histological structure of the central and portal area of the liver (Figures 7A,D). The OVX group showed focal necrosis surrounding the central vein and fatty degeneration surrounding the hepatic portal area (Figure 7B,E). The OVX/*D* group demonstrated normal histological structure of the central and portal area of the liver (Figures 7C,F). Liver-lesion scores (Table 8) were significantly increased following ovariectomy. The OVX group showed the highest scores for hepatic steatosis, necrosis, inflammatory-cell infiltration, and vascular congestion. Treatment with *D. costus* significantly reduced each lesion score and lowered the total liver-lesion score by approximately 64% relative to untreated OVX rats. However, the treated group retained higher lesion scores than the sham group, indicating partial histological improvement.

**FIGURE 7:**
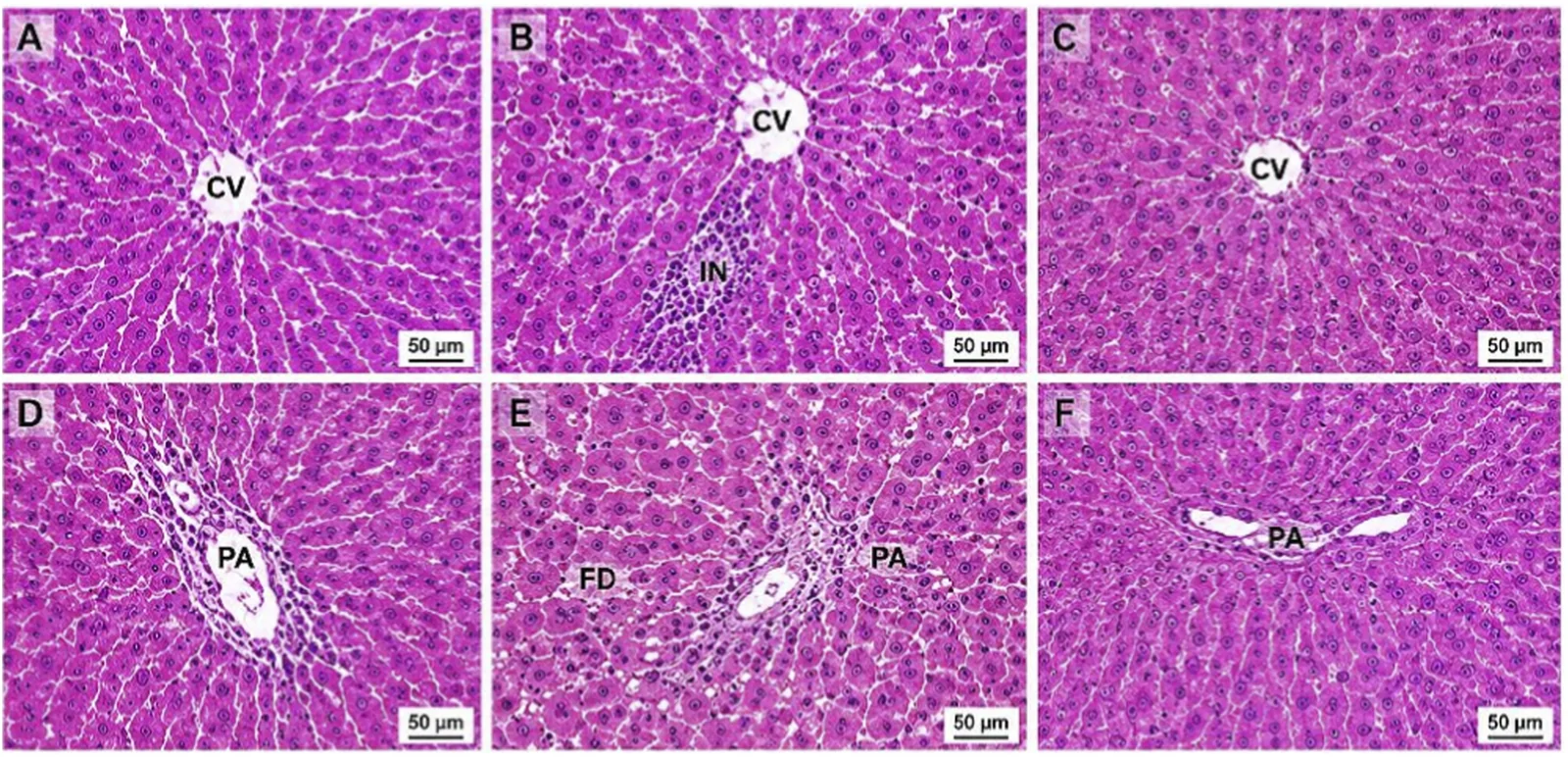
Photomicrographs of H&E-stained liver sections demonstrated that **(A,D)** sham groups exhibited normal hepatic architecture with intact central veins (CV) and portal areas (PA). **(B,E)** OVX groups displayed pathological changes including focal pericentral necrosis (N) and periportal fatty degeneration (FD). **(C,F)** OVX/*D*-treated livers showed restored histology with preservation of CV and PA architecture, comparable to that of sham operated rats (400X).

**TABLE 8:** Semiquantitative hepatic-lesion scores in sham-operated, ovariectomized (OVX), and *Dolomiaea costus (*Syn*. Saussurea costus)*-treated female rats.

Liver parameter, score 0–4	Sham	OVX	OVX/D
Hepatic steatosis	0.31 ± 0.20^c^	3.21 ± 0.31^a^	1.21 ± 0.21^b^
Hepatocellular necrosis	0.21 ± 0.13^c^	2.81 ± 0.31^a^	0.91 ± 0.21^b^
Inflammatory-cell infiltration	0.31 ± 0.20^c^	2.50 ± 0.30^a^	1.01 ± 0.21^b^
Sinusoidal/vascular congestion	0.20 ± 0.11^c^	2.31 ± 0.31^a^	0.81 ± 0.20^b^
Total liver-lesion score, 0–16	1.00 ± 0.41^c^	10.81 ± 0.80^a^	3.92 ± 0.63^b^

Data are presented as mean ± SE, with n = 6 animals per group. Steatosis, hepatocellular necrosis, inflammatory-cell infiltration, and sinusoidal or vascular congestion were scored from 0 to 4 according to lesion severity. The total lesion score ranged from 0 to 16. Group comparisons were analyzed using the non-parametric Kruskal–Wallis test followed by Dunn’s post-hoc test with Holm adjustment for multiple comparisons. Different superscript letters within the same row indicate statistically significant differences among groups at *p* < 0.05. OVX, ovariectomized group; OVX/D, ovariectomized group administered 300 mg/kg body weight of *Dolomiaea costus* (Syn. *Saussurea costus*) roots extract.

#### 3.6.3 Histomorphometric and immunohistochemical findings

Histomorphometric and immunohistochemical analyses revealed marked alterations in adipose tissue, uterine architecture, and femoral bone following ovariectomy, whereas treatment with *D. costus* attenuated several of these changes (Table 9).

**TABLE 9:** Histomorphometric and immunohistochemical measurements in sham-operated, ovariectomized (OVX), and *Dolomiaea costus*-treated female rats.

Parameter	Sham	OVX	OVX/*D*
Adipocyte diameter (µm)	26.01 ± 9.02^c^	50.00 ± 14.06^a^	35.10 ± 8.14^b^
Adipocyte area (µm^2^)	531.34 ± 368.52^c^	1963.50 ± 1,104.27^a^	967.62 ± 448.80^b^
TNF-α-positive area (%)	22.0 ± 5.0^c^	68.0 ± 13.0^a^	36.0 ± 6.0^b^
Endometrial thickness (µm)	285.01 ± 20.02^a^	150.05 ± 10.03^b^	170.03 ± 15.01^b^
Myometrial thickness (µm)	100.06 ± 8.02^a^	45.01 ± 5.03^b^	50.02 ± 6.04^b^
Perimetrial thickness (µm)	45.02 ± 5.04^b^	65.04 ± 5.05^a^	50.07 ± 5.09^ab^
Uterine ER-α-positive area (%)	57.05 ± 8.05^a^	20.03 ± 8.06^c^	40.04 ± 6.02^b^
Mean femoral trabecular thickness (µm)	92.21 ± 6.01^a^	46.11 ± 4.15^c^	73.54 ± 5.25^b^
Trabecular area fraction (%) of the femur	48.25 ± 3.11^a^	23.12 ± 2.21^c^	38.15 ± 3.12^b^
Trabecular number, Tb.N (mm^-1^)	4.21 ± 0.28^a^	2.12 ± 0.19^c^	3.37 ± 0.24^b^
Trabecular separation, Tb.Sp (µm)	145.32 ± 11.64^c^	318.46 ± 22.15^a^	205.73 ± 16.82^b^
Osteoblast surface, Ob.S/BS (%)	23.82 ± 2.11^a^	11.43 ± 1.32^c^	18.91 ± 1.67^b^
Osteoclast surface, Oc.S/BS (%)	5.76 ± 0.72^c^	16.54 ± 1.48^a^	9.31 ± 0.91^b^

Data are presented as mean ± SE, with n = 6 animals per group. Three sections were examined per animal, and measurements from the evaluated fields and sections were averaged to obtain one animal-level value. Group comparisons were analyzed using the non-parametric Kruskal–Wallis test followed by Dunn’s post-hoc test with Holm adjustment for multiple comparisons. Different superscript letters within the same row indicate statistically significant differences among groups at *p* < 0.05. OVX, ovariectomized group; OVX/D, ovariectomized group administered 300 mg/kg body weight of *Dolomiaea costus* (Syn. *Saussurea costus*) roots extract.

Ovariectomy induced pronounced adipocyte hypertrophy. The mean adipocyte diameter increased significantly from 26.01 ± 9.02 µm in the sham group to 50.00 ± 14.06 µm in the OVX group (p < 0.05). Similarly, adipocyte area increased from 531.34 ± 368.52 µm^2^ to 1963.50 ± 1,104.27 µm^2^. Administration of *D. costus* significantly reduced the adipocyte diameter and area to 35.10 ± 8.14 µm and 967.62 ± 448.80 µm^2^, respectively, compared with that in untreated OVX rats, although both parameters remained significantly higher than those of sham animals (p < 0.05). Consistent with these morphological changes, TNF-α immunoreactivity was markedly enhanced following ovariectomy, with the TNF-α-positive area increasing from 22.0% ± 5.0% in sham rats to 68.0% ± 13.0% in OVX rats. *D. costus* treatment significantly reduced TNF-α-positive staining to 36.0% ± 6.0% (p < 0.05), indicating the attenuation of the OVX-associated inflammatory response.

Marked uterine structural alterations were also observed after ovariectomy. Endometrial thickness decreased significantly from 285.01 ± 20.02 µm in the sham group to 150.05 ± 10.03 µm in OVX rats (*p* < 0.05). The corresponding value in the OVX/*D. costus* group was 170.03 ± 15.01 µm. Although this represented a numerical increase relative to untreated OVX rats, the difference between these two groups was not statistically significant, and both remained significantly lower than the sham group. A similar pattern was evident for myometrial thickness, which declined from 100.06 ± 8.02 µm in sham animals to 45.01 ± 5.03 µm after ovariectomy. Treatment with *D. costus* led to a value of 50.02 ± 6.04 µm, which did not differ significantly from that of the untreated OVX group. In contrast, perimetrial thickness increased significantly after ovariectomy, from 45.02 ± 5.04 µm in sham rats to 65.04 ± 5.05 µm in OVX rats (*p* < 0.05). The OVX/*D. costus* group exhibited an intermediate value of 50.07 ± 5.09 µm that was not significantly different from either the sham or OVX groups.

Immunohistochemical analysis of uterine estrogen receptor-α (ER-α) demonstrated a substantial reduction in receptor expression following ovariectomy. The ER-α-positive area decreased from 57.05% ± 8.05% in sham rats to 20.03% ± 8.06% in OVX animals (*p* < 0.05). Treatment with *D. costus* significantly increased ER-α immunopositivity to 40.04% ± 6.02% compared with untreated OVX rats; however, the value remained significantly below that recorded in the sham group (*p* < 0.05).

Femoral histomorphometry further demonstrated pronounced OVX-induced deterioration of trabecular bone architecture. The mean trabecular thickness decreased from 92.21 ± 6.01 µm in sham rats to 46.11 ± 4.15 µm in OVX rats, while the trabecular area fraction declined from 48.25% ± 3.11% to 23.12% ± 2.21% (*p* < 0.05). Trabecular number was likewise significantly reduced from 4.21 ± 0.28 mm^-1^ to 2.12 ± 0.19 mm^-1^. Conversely, trabecular separation increased markedly from 145.32 ± 11.64 µm in sham animals to 318.46 ± 22.15 µm following ovariectomy. Administration of *D. costus* significantly improved all of these trabecular indices, increasing trabecular thickness, area fraction, and number to 73.54 ± 5.25 µm, 38.15% ± 3.12%, and 3.37 ± 0.24 mm^-1^, respectively, while reducing trabecular separation to 205.73 ± 16.82 µm. Nevertheless, these parameters remained significantly different from sham values (*p* < 0.05), indicating partial rather than complete restoration of trabecular architecture.

Changes in bone cellular surfaces were consistent with the structural findings. Osteoblast surface (Ob.S/BS) decreased significantly from 23.82% ± 2.11% in sham animals to 11.43% ± 1.32% in OVX rats, whereas osteoclast surface (Oc.S/BS) increased from 5.76% ± 0.72% to 16.54% ± 1.48% (*p* < 0.05). *D. costus* treatment significantly increased osteoblast surface to 18.91% ± 1.67% and reduced osteoclast surface to 9.31% ± 0.91% compared with that in untreated OVX rats. However, both indices remained significantly different from sham levels.

An integrated overview of the principal study findings is presented in Table 10. Ovariectomy was associated with adverse changes across body-weight, adiposity, lipid, glycemic, adipokine, antioxidant, uterine, adipose-tissue, femoral, and hepatic outcomes. Administration of *D. costus* extract was associated with improvement in most measured outcomes relative to untreated OVX animals. However, several variables remained different from those of the sham group, indicating partial rather than complete recovery.

**TABLE 10:** Integrated summary of the effects of ovariectomy and treatment with *Dolomiaea costus (*Syn*. Saussurea costus*) roots extract.

Outcome domain	Effect observed in OVX animals relative to sham	Effect of *D. costus* relative to untreated OVX animals	Evidence type
Final body weight and weight gain	Increased	Reduced toward sham values	Quantitative
Absolute adipose-tissue weight	Increased	Reduced	Quantitative
Relative adipose-tissue weight	Increased	Reduced	Quantitative
Total cholesterol and triglycerides	Increased	Reduced	Quantitative
HDL-C	Decreased	Increased toward sham values	Quantitative
Fasting glucose and HOMA-IR	Increased	Reduced	Quantitative
Serum insulin	Slightly decreased	Increased toward the sham value	Quantitative
Albumin and total protein	Decreased	Partially increased	Quantitative
Total and ionized calcium	Decreased	Partially increased	Quantitative
Resistin and leptin	Increased	Reduced	Quantitative
Adiponectin	Decreased	Increased	Quantitative
Total antioxidant capacity	Decreased	Increased toward the sham value	Quantitative
Endometrial thickness	Decreased	Increased	Quantitative histomorphometry
Myometrial thickness	Decreased	Increased	Quantitative histomorphometry
Perimetrial thickness	Increased	Reduced toward the sham value	Quantitative histomorphometry
Uterine-gland number or density	Decreased	Increased	Quantitative histomorphometry
Uterine ER-α-positive area	Decreased	Partially increased	Quantitative IHC
Mean adipocyte diameter	Increased	Reduced	Quantitative histomorphometry
Adipose-tissue TNF-α-positive area	Increased	Reduced	Quantitative IHC
Femoral trabecular architecture	Apparently thinner and more disrupted trabecular profiles	Apparently improved trabecular organization	Qualitative histology
Hepatic architecture	Focal pericentral necrosis and periportal fatty degeneration	Fewer apparent degenerative alterations	Qualitative histology

The table presents an integrated directional summary of the principal study findings. Detailed numerical values, measures of variability, overall statistical tests, and post hoc comparisons are reported in the corresponding Results tables and figures. “Increased” and “decreased” describe statistically supported group differences for quantitative outcomes. Femoral and hepatic observations are identified as qualitative because bone-mineral-density measurements, biomechanical testing, systematic trabecular histomorphometry, and validated liver-lesion scoring were not performed. ER-α, estrogen receptor alpha; HDL-C, high-density lipoprotein cholesterol; HOMA-IR, homeostatic model assessment of insulin resistance; IHC, immunohistochemistry; OVX, ovariectomized; TNF-α, tumor necrosis factor alpha.

## 4 Discussion

In this study, we comprehensively investigate the phytochemical composition and therapeutic potential of *D. costus* roots extract, particularly its efficacy in mitigating postmenopausal osteoporosis and metabolic dysregulation in OVX rats. The findings reveal significant biochemical, hormonal, and histological improvements, supported by robust phytochemical characterization.

Our quantitative analysis revealed that *D*. *costus* crude extract possesses considerable total phenolic content and flavonoid content. These findings are consistent with the work done by Kanmani Bharathi and Prakash (2025), who demonstrated high polyphenolic concentrations within various species belonging to the Asteraceae family. Sesquiterpene lactones (e.g., dehydrocostuslactone in unsaponifiable fraction) and chlorogenic acid (via HPLC) are of particular note, corroborating studies identifying these compounds as key bioactive constituents in Asteraceae species (Moujir et al., 2020). Furthermore, chlorogenic acid was found at measurable amounts via HPLC-DAD validation; this aligns closely with prior reports listing it among dominant bioactive constituents conferring health benefits including potent antioxidative properties (Hu et al., 2024). The use of multiple chemical assays provided complementary information about the chemical antioxidant properties of the extract. DPPH and ABTS demonstrated concentration-dependent radical-scavenging activity under different reaction conditions, whereas FRAP and phosphomolybdenum assays reflected electron-donating and reducing capacity. The extract generally showed lower activity than the pure reference antioxidants, supporting its characterization as having moderate, assay-dependent chemical antioxidant capacity rather than potent antioxidant activity.

These cell-free assays characterize the direct chemical reactivity of the extract under controlled experimental conditions. They do not account for gastrointestinal absorption, metabolism, systemic exposure, tissue distribution, or interactions with endogenous antioxidant systems. Therefore, they cannot independently demonstrate *in vivo* antioxidant efficacy or explain the higher serum total antioxidant capacity observed in the extract-treated ovariectomized rats. The chemical extract assays and serum TAC results are interpreted as complementary but analytically and biologically distinct findings. Pharmacokinetic studies and measurements of specific oxidative-stress biomarkers and antioxidant enzymes would be required to establish whether absorbed extract constituents directly influenced systemic redox regulation. DPPH chemical IC_50_ value is considerably higher than pure standards such as BHT or vitamin C, yet it is sufficient for biological efficacy given the complex plant matrixes involved (López-Alarcón and Denicola, 2013). DPPH/ABTS-neutralization capacities proportional not only to TPC/TFC values but also synergistic effects imparted from minor compounds detected through GC–MS profiling including sesquiterpene lactones such as dehydrocostuslactone were observed in the present study. Phenolics and sesquiterpenes mitigate oxidative stress by chelating metals in FRAP assay and quenching free radicals (DPPH/ABTS), thereby reducing tissue damage (Moukette et al., 2015). Comprehensive chemical characterization using GC–MS expanded upon existing knowledge regarding unsaponifiable/saponifiable fractions that identify numerous terpenoid moieties exclusive or highly enriched in *D. costus* roots. GC–MS data match reports identifying dehydrocostuslactone as a major anti-inflammatory compound in *D. costus* (Kaur et al., 2025). GC–MS library comparison provisionally annotated several constituents in the saponifiable and unsaponifiable fractions. Dehydrocostuslactone was among the prominent tentatively annotated sesquiterpene lactones, whereas chlorogenic acid was identified and quantified by HPLC-DAD using an authentic reference standard. Because the GC–MS assignments were not confirmed by authentic standards, it should be interpreted as provisional chemical annotations. Consequently, the biological effects observed in the animal experiment cannot be attributed to any individual GC–MS peak.

Ovarian hormone deprivation models typically recapitulate critical aspects observed clinically during menopause, namely, increased body fat accumulation (dyslipidemia) along with persistent hyperglycemia caused partly due to peripheral insulin resistance development (Porada et al., 2023). Our intervention significantly ameliorated these elevations, seen notably when comparing OVX versus OVX/*D* groups across final weight/fat mass gains paralleling improved serum lipid profile indices and marked reductions observed in both TG/TC levels while elevating HDL concentration comparable almost returning baseline control states and mirroring studies where sesquiterpene lactones suppressed adipogenesis via PPAR-γ inhibition (Feng et al., 2016). Similar anti-obesity/lipid lowering trends have been described recently concerning other phytoestrogen-rich herbal extracts used against estrogen withdrawal syndromes (Myasoedova et al., 2016).

The metabolic profile changes observed in the OVX rats mirror key clinical features of menopause-induced metabolic syndrome. The significant decrease in serum insulin levels coupled with compensatory hyperglycemia and heightened HOMA-IR indices indicates substantial impairment of insulin secretion and peripheral tissue resistance, a hallmark phenomenon following ovarian hormone withdrawal (Houston and Templeman, 2025). A sharp, disproportionate spike in fasting blood glucose outweighs the drop in circulating insulin (Inchauspé, 2022; Valent and Barbour, 2025). Estrogen modulates glucose homeostasis through direct actions on pancreatic β-cells and by enhancing systemic insulin sensitivity; thus, its depletion leads not only to increased hepatic gluconeogenesis but also diminished GLUT4-mediated glucose uptake within adipose depots (Hanhineva et al., 2010). Crucially, treatment with *D. costus* root extract effectively normalized these parameters. The fasting blood glucose was reduced while both circulating insulin concentrations and whole-body HOMA-IR values significantly improved when compared to that in untreated ovariectomized controls. This indicates a restoration of functional crosstalk between pancreatic function and target tissues, at least partly due to bioactive phytochemicals. Chlorogenic acid, a major phenolic acid found in the present study, is linked to improved insulin sensitivity and lipid metabolism in osteoporosis models, supporting our biochemical findings (Yan et al., 2022). It may influence PPARγ signaling pathways, which are known for their involvement in promoting adiponectin production and improving the overall glycemic control (Zhao et al., 2016). Histological data further corroborate this biochemical normalization. The white adipose deposits from OVX animals exhibited classic signs associated with pathological expansion, including hypertrophy, increased volume per cell, and prominent vascular dilatation, which indicates local inflammation or hypoxic stress induced via excessive lipid accumulation, a process shown previously by Trayhurn and Alomar (2015) during obesity progression. Conversely, the OVX/*D* group maintained normally sized fat cells, reminiscent of and structurally and phenotypically similar to those found within intact sham-operated cohorts, which is indicative again that estrogen-mimicking compounds counteract maladaptive postmenopausal remodeling seen most starkly under hypoestrogenic states (Alizadeh et al., 2023). Immunohistochemical analysis supported these findings. Marked overexpression of TNF-α was detected surrounding adipocyte interfaces uniquely among the OVX group, indicating a pro-inflammatory microenvironment favoring cellular infiltration, apoptosis linked ultimately toward worsening aberrant metabolism, and bone turnover risk profiles after menopause onset (Duarte Mendes et al., 2023). Notably, however, such upregulation became markedly attenuated upon administration, stunningly mirroring the expression patterns typically attributed to eugonadal reference standards, that is, sham or *D. costus*-only test arms, which collectively support published work demonstrating potent anti-inflammatory effects imparted mainly through sesquiterpene lactones, flavonoid-rich phytoextracts targeting the NF-ĸB pathway inhibition downstream cytokine regulation activity (Gahtori et al., 2023).

In the adipokine analyses, the OVX/*D* group notably decreased leptin/resistin alongside elevated adiponectin levels, reinforcing long-held views proposing the beneficial impact exerted downstream via PI3K/Akt/mTOR cascades critically controlling the central and peripheral energy, the adiposity regulation under oxidative stress challenge scenarios induced prematurely lost gonadal feedback inhibition cycle (Rak et al., 2017). The OVX group’s elevated leptin and resistin levels correlate mechanistically with bone loss through multiple pathways. Leptin overexpression stimulates osteoclast differentiation via indirect CNS-mediated RANKL upregulation in stromal cells (Otto et al., 2020), while resistin directly enhances NF-κB signaling to promote osteoclastic bone resorption (Abu-Amer, 2013). Conversely, the OVX/*D* group restored adiponectin levels that exert dual osteoprotective effects, suppressing osteoclastogenesis through OPG/RANKL axis modulation while enhancing Wnt/β-catenin signaling for osteoblast mineralization (Carrillo-López et al., 2021). The parallel TAC improvement directly combats oxidative inhibition of osteoblastic alkaline phosphatase activity, as ROS scavenging preserves Runx2 transcriptional competence for bone matrix deposition (Li et al., 2025). These coordinated changes explain the histological reversal of OVX-induced trabecular thinning observed in the study. Clinical studies further support the adipokine–osteoporosis linkage, showing that serum resistin levels inversely correlate with lumbar spine BMD in postmenopausal women (Tariq et al., 2021). The extract’s sesquiterpene lactones (Siddiqui et al., 2024) likely disrupt this pathological loop through PPARγ antagonism and reduce marrow adipocyte expansion while redirecting mesenchymal stem-cell differentiation toward osteoblasts rather than adipocytes (Schilling et al., 2014), as evidenced by histological examination of the transverse sections of the femur bone.

The significantly decreased serum albumin levels, total protein, total calcium, and ionized calcium observed in the OVX group are consistent with established consequences of estrogen deficiency following ovariectomy. Estrogen is pivotal in bone metabolism, maintains overall protein synthesis within hepatic tissues, and regulates mineral homeostasis (Palmisano et al., 2017). Its depletion accelerates bone turnover, favoring resorption over formation, which leads directly to mobilization and loss of skeletal stores, contributing to hypocalcemia commonly seen both clinically among menopausal women and experimentally after ovariectomy (Cheng et al., 2022).

Lower albumin concentrations further compromise systemic calcemic balance since approximately 40%–45% of circulating calcium remains bound tightly to plasma proteins such as albumin (Levitt and Levitt, 2016). Therefore, hypoalbuminemia reduces this reservoir capacity for buffering acute shifts between free and thus biologically active ionized forms versus inactive fractions; ultimately exacerbating functional deficits when dietary intake or parathyroid compensation proves inadequate (Idasiak-Piechocka et al., 2025). Additionally, suppressed liver-synthesized globulins and total proteins could reflect either diminished synthetic drive under chronic low-grade inflammatory states or direct hepatocellular dysfunction induced by increased oxidative stress associated with ovarian hormone deprivation (Song et al., 2025). However, *D. costus* extract intervention led to marked improvement across these parameters. Treated animals approached sham controls regarding restored total calcium and ionized calcium status, accompanied by normalization trends toward higher serum proteomic indices. This effect may be attributed primarily to several bioactive components identified within *D. costus*, including chlorogenic acid as reported previously for their antioxidative properties that protect against tissue catabolism while simultaneously upregulating osteoblastic differentiation/survival through modulation pathways involving ERK/p38 MAP kinases that are essential for matrix organization/mineral deposition (Nguyen et al., 2024). These data support previous work that demonstrated enhanced intestinal absorption reduced urinary excretion rates linked specifically to plant-derived terpenoids’ vitamin D-agonist activity (Gundala and Aneja, 2014), a plausible explanation here considering our GC–MS analysis highlighting high sesquiterpene lactone content capable of mimicking classical steroid actions upon target organs involved in mineral reabsorption/homeostatic control mechanisms (Paço et al., 2022).

Uterus atrophy, glandular reduction, and ER depletion in OVX rats were ameliorated, indicating phytoestrogenic activity. *D. costus* crude extract treatment restored the endometrial thickness and ER-α expression, consistent with prior work on sesquiterpenes as selective ER modulators. Similar to *Cimicifuga racemosa* (black cohosh) (Gaube et al., 2007), *D. costus* exhibits phytoestrogenic properties, offering a safer alternative to hormone therapy (Moreira et al., 2014). However, no histological evidence of hyperplasia was observed under the present experimental conditions. Moreover, OVX-induced trabecular thinning and marrow adiposity were reversed by *D. costus*, indicating osteoprotection. This aligns with dehydrocostus lactone’s reported stimulation of osteoblast differentiation (Choi et al., 2009). OVX-induced necrosis and steatosis were prevented by *D. costus*, underscoring the hepatoprotective effects of flavonoids and sesquiterpenes (Zárybnický et al., 2018).

The hepatic alterations observed following ovariectomy are consistent with previous reports of Yu et al. (2022) linking hypoestrogenism to increased vulnerability of hepatic tissues toward metabolic derangements and injury. The OVX group’s presentation on focal necrosis surrounding central veins alongside marked fatty degeneration reflects a well-documented postmenopausal pattern wherein the withdrawal of ovarian hormones disrupts lipid metabolism homeostasis within hepatocytes (Barsalani, 2010). Estrogens play a hepatoprotective role against steatohepatitis through dual mechanisms: upregulation of PPARα-mediated fatty acid β-oxidation and suppression of lipogenic transcription factors. Estrogen deficiency consequently promotes hepatic lipid accumulation through impaired fat metabolism while simultaneously creating a pro-inflammatory microenvironment characterized by elevated oxidative stress, cytokine release, and enhanced apoptotic signaling pathways (Morán-Costoya et al., 2021).

The administration of *D*. *costus* extract led to near-complete normalization of livers from treated subjects that exhibited preserved architecture across portal tracts and central vascular regions indistinguishable from sham specimens according to our H&E-based assessment. This significant reversal can likely be attributed primarily to several bioactive constituents identified through phytochemical profiling, notably sesquiterpene, lactones, flavonoids, or chlorogenic acids, which collectively protect membranes by scavenging ROS while concurrently inhibiting pro-inflammatory mediators such as TNF-α/NF-ĸB that are seen to be upregulated under extreme metabolic duress (Elnour and Abdurahman, 2024). Recent studies reinforce these observations by documenting plant-derived polyphenols’ efficacy in preventing mitochondrial dysfunction and fibrotic transformation that are characteristic of model systems simulating menopause-like endocrine status loss (Ricci, 2012). Together, this indicates that *D. costus* root may offer critical multifactorial protection during periods of high risk associated with chronic low-grade inflammation/lipid overload, which are typical of menopausal transition states.

The observed biochemical and histological differences may involve several interconnected pathways, but these mechanisms were not directly examined. Estrogen-responsive signaling can influence adipose distribution, inflammatory activity, and bone turnover, whereas NF-κB-associated signaling contributes to inflammatory cytokine expression and osteoclast differentiation. The RANK/RANKL/OPG system and Wnt/β-catenin signaling regulate the balance between bone resorption and formation, while AMPK and PPARγ participate in energy metabolism and adipocyte differentiation. Some metabolites reported in *D. costus*, including dehydrocostus lactone and costunolide, have demonstrated activities involving selected pathways in other experimental systems. Nevertheless, in the present study, we did not measure receptor binding, phosphorylated signaling proteins, gene expression, RANKL, OPG, AMPK, PPARγ, or Wnt/β-catenin. These pathways, therefore, provide testable hypotheses for future studies rather than confirmed explanations of the current findings.

Several limitations should be acknowledged. First, only a single dose of the extract was evaluated, precluding the assessment of dose–response relationships and determination of the minimum effective or optimal dose. In addition, the study did not include an estradiol-, raloxifene-, or standard hormone-therapy-treated positive-control group; therefore, the effects of the *D. costus* extract cannot be directly compared with those of established interventions. Second, although uterine ER-α immunoreactivity was quantified, increased receptor staining does not establish receptor binding, transcriptional activation, or direct estrogen-receptor agonism. Receptor-binding assays, reporter-gene studies, Western blotting, qPCR analysis of estrogen-responsive genes, and other functional estrogen-response assays were not performed. Accordingly, the findings should not be interpreted as definitive evidence of direct phytoestrogenic activity. Third, several signaling pathways discussed as potential mechanisms were not directly examined. In particular, RANKL, OPG, NF-κB, AMPK, PPARγ, Wnt/β-catenin, and related molecular intermediates were not measured. Consequently, the proposed antioxidant, anti-inflammatory, anti-adipogenic, and bone-protective mechanisms remain inferential and require direct experimental validation. Fourth, the skeletal evaluation did not include bone-mineral-density measurements, biomechanical testing, or comprehensive dynamic bone histomorphometry. Therefore, the histological and biochemical findings cannot establish improvements in bone strength or fracture resistance. Fifth, the chemical analyses characterized the extract as a complex mixture but did not identify the individual metabolite or combination of metabolites responsible for the observed biological effects. Several GC–MS constituents were tentatively assigned by comparison with Wiley and NIST mass-spectral libraries without confirmation using authentic standards or formal retention-index determination. Unusual or low-confidence assignments should, therefore, be interpreted cautiously. Moreover, chromatographic peak-area percentages represent relative abundance rather than absolute constituent concentrations. Sixth, pharmacokinetics, oral bioavailability, systemic exposure, active-metabolite profiles, residual-solvent concentrations, and comprehensive sub-chronic reproductive and organ toxicity were not evaluated. Thus, conclusions regarding effective systemic exposure and long-term safety cannot be drawn. Finally, the ovariectomized-rat model reproduces selected consequences of estrogen deficiency but does not encompass the physiological and clinical complexity of natural human menopause. The findings should, therefore, be regarded as preliminary preclinical evidence requiring independent dose-ranging, mechanistic, pharmacokinetic, and toxicological confirmation before the therapeutic efficacy or safety in menopausal women can be inferred.

## 5 Conclusion

In ovariectomized rats, oral administration of the chemically characterized *D*. *costus* root extract was associated with improvements in selected metabolic, inflammatory, antioxidant, skeletal, hepatic, and uterine outcomes compared with untreated ovariectomized animals. The detected sesquiterpene lactones and phenolic metabolites provide a plausible chemical context for the observed findings. However, the present study cannot identify the active metabolites, demonstrate direct estrogen-receptor agonism, or establish a causal molecular mechanism. Further investigations should include standardized extract replication, dose–response assessment, pharmacokinetic studies, comprehensive toxicological evaluation, receptor-based functional assays, and confirmation in independent experimental models. Clinical efficacy and safety in menopausal women cannot be inferred from the current animal study.

## Data Availability

The raw data supporting the conclusions of this article will be made available by the authors, without undue reservation.
